# Hydrogel-Based Mechanical Sensors for Sleep Respiratory Monitoring: Recent Advances and Future Perspectives

**DOI:** 10.3390/s26175642

**Published:** 2026-09-04

**Authors:** Yuanfeng Sun, Liangchao Li, Yuan Shi, Jian Jiao, Taomei Li, Xiangdong Tang, Yihao Long, Liang He, Lan Zhang

**Affiliations:** 1 Sleep Medicine Center, West China Hospital, Sichuan University, Chengdu 610041, China; sunny@wchscu.edu.cn (Y.S.); shiyuan@wchscu.cn (Y.S.); jiaojian@wchscu.edu.cn (J.J.); maytaomeili@hotmail.com (T.L.); tangxiangdong@scu.edu.cn (X.T.); 2 School of Mechanical Engineering, Sichuan University, Chengdu 610065, China; liliangchao1127@163.com (L.L.); longyihao17@stu.scu.edu.cn (Y.L.); hel20@scu.edu.cn (L.H.); 3 Mental Health Center, West China Hospital, Sichuan University, Chengdu 610041, China; 4 National Center for Mental Disorders, West China Hospital, Sichuan University, Chengdu 610041, China

**Keywords:** hydrogel-based mechanical sensors, sleep respiratory, hydrogel-based composites

## Abstract

**Highlights:**

1. Sensing mechanisms, materials, and fabrication strategies of hydrogel-based mechanical sensors are systematically reviewed.
2. Critical challenges, including high accuracy, rapid response, long-term stability, and high wearing comfort, are summarized and discussed.

**What are the main findings?**

- Recent advances in hydrogel-based mechanical sensors for sleep respiratory monitoring are systematically summarized.
- The relationships among sensing mechanisms, materials, fabrication strategies, and sensing performance are comprehensively discussed.

**What are the implications of the main findings?**

- Current challenges and optimization strategies for improving sensor performance are summarized.
- This review provides theoretical guidance and research references for the design and clinical translation of high-performance hydrogel-based mechanical sensors.

**Abstract:**

Sleep respiratory monitoring plays a crucial role in the early diagnosis and health management of sleep-related respiratory disorders. However, conventional monitoring devices often suffer from some critical issues, such as poor wearing comfort and insufficient capability for long-term continuous monitoring, hindering their applications in non-invasive and prolonged sleep monitoring. In recent years, hydrogel-based mechanical sensors have attracted increasing attention for sleep respiratory monitoring owing to their outstanding flexibility, biocompatibility, mechanical compatibility with biological tissues, and excellent sensing performance, demonstrating great potential for practical applications. A comprehensive overview of recent advances in hydrogel-based mechanical sensors for sleep-related respiratory monitoring is provided in this review. The sensing mechanisms, materials, fabrication strategies, and representative applications are systematically summarized, with particular emphasis on the key factors governing sensor performance and their translation toward practical use. Furthermore, current challenges and corresponding optimization strategies are discussed. This review aims to provide valuable insights into the rational design and optimization of high-performance hydrogel-based mechanical sensors, and important strategies for the further development of advanced technologies for sleep-related respiratory monitoring.

## 1. Introduction

Sleep is an important physiological process, playing an essential role in maintaining normal bodily functions and overall health, while sleep-related breathing disorders have become a significant global public health concern. Obstructive sleep apnoea (OSA) is considered as the most prevalent sleep-related breathing disorder, featured by recurrent episodes of upper airway obstruction during sleep. It can induce intermittent hypoxia, sleep fragmentation, and autonomic dysfunction, thereby increasing the risk of various chronic diseases, including hypertension, cardiovascular disease, diabetes, and neurodegenerative disorders. Owing to the concealed nature of OSA symptoms and the high rate of underdiagnosis, the development of long-term, continuous, and non-invasive sleep respiratory monitoring technologies is of great importance for early screening, clinical diagnosis, and evaluation of therapeutic outcomes [1,2].

Currently, polysomnography remains the clinical gold standard for diagnosing sleep-related breathing disorders. However, its dependence on complex electrode configurations, specialized equipment, and clinical environments, results in several limitations, including high cost, complicated operation, poor patient comfort, and limited feasibility for long-term home-based monitoring [3]. In recent years, wearable flexible sensors are emerging as highly promising devices for sleep respiratory monitoring, owing to their lightweight characteristics, mechanical flexibility, and capability for continuous physiological signal acquisition [4]. Among various types of flexible sensors, mechanical sensors can directly capture thoracic and abdominal movements, airflow-induced changes, and tissue deformation during respiration, enabling them well suited for sleep respiratory monitoring [5].

Among the diverse materials developed for flexible sensors, hydrogels have attracted extensive attention as promising platforms for wearable mechanical sensing systems, due to their excellent flexibility and biocompatibility, high water content, and mechanical performance similar to those of human soft tissues. Through the construction of electronically conductive networks, ionically conductive pathways or hybrid electron-ion conductive systems, hydrogel-based mechanical sensors can effectively transform subtle mechanical deformations into measurable electrical signals [6,7,8]. Moreover, the incorporation of functional components, such as MXene, graphene, liquid metals, and ionic liquids, together with advanced structuring strategies, including multi-crosslinking networks, double-network architectures, and biomimetic designs, has substantially improved the sensitivity, stability, comfort, and long-term wearability of hydrogel-based mechanical sensors. These advances have opened new opportunities for their applications in sleep respiratory monitoring [9,10].

In recent years, there is rapid development in flexible hydrogel-based mechanical sensors. Previous reviews have mainly focused on flexible electronics, bioelectronic interfaces, hydrogel-based materials, or wearable health monitoring technologies, providing comprehensive discussions on conductivity related mechanisms, materials, and device performance [11,12,13]. However, systematic reviews specifically targeting hydrogel-based mechanical sensors for sleep apnoea monitoring are few, particularly those addressing the relationships among sensing mechanisms, materials investigation, structural designs, and performance optimization strategies [4]. In addition, the intrinsic connections between conductivity related mechanisms, materials, and fabrication approaches have not been fully elucidated. Key challenges associated with long-term wearable applications, including comfort, monitoring reliability, and intelligent signal analysis, also require further investigation.

In this context, this review summarizes the recent progresses in hydrogel-based mechanical sensors for sleep apnoea monitoring. First, the fundamental sensing mechanisms of hydrogel-based mechanical sensors are systematically elaborated. Subsequently, various conductive systems and structural design strategies are discussed in terms of materials and fabrication methods. Finally, recent applications in sleep respiratory monitoring are comprehensively reviewed, followed by an analysis of current challenges and corresponding optimization strategies, involving sensitivity, long-term stability, environmental adaptability, wearing comfort, and analysis of intelligent technologies. This review aims to provide insights into the rational design of high-performance hydrogel-based mechanical sensors and promote their future development toward practical and clinical sleep respiratory monitoring applications.

## 2. Hydrogel-Based Mechanical Sensors

### 2.1. Fundamentals of Hydrogel Network Structures and Force Sensing

Hydrogels are macromolecular materials composed of three-dimensional polymer networks with high water content. Their mechanical behaviour is primarily governed by network topology, cross-linking density, segment–segment interactions, and inter-network interactions. In mechanosensitive hydrogels, the network structure not only determines the elastic modulus, deformability, toughness, and energy dissipation capacity, but also regulates the transmission of mechanical stimuli and the dynamic evolution of conductive pathways, thereby affecting sensing sensitivity, detection range, and cycling stability [14].

Based on the cross-linking mechanism, hydrogel networks can be classified as physically, chemically, or dynamically cross-linked. Physical cross-linking primarily relies on reversible non-covalent interactions, such as hydrogen bonding, electrostatic interactions, and hydrophobic interactions, providing good dynamic reconfiguration and high energy dissipation [15]. Chemical cross-linking forms three-dimensional networks through stable covalent bonds, offering high structural stability and dimensional retention. However, damage accumulated under continuous mechanical loading is generally difficult to be repaired [16]. Dynamic cross-linking, based on reversible interactions such as dynamic covalent bonds and metal coordination, combines structural stability with dynamic reconfiguration, is helpful in balancing mechanical strength, toughness, and fatigue resistance [17]. These cross-linking strategies provide the fundamental basis for tailoring the mechanical performance and sensing responses of hydrogels.

Multinetwork structures further expand the possibilities for regulating hydrogel performance. Double-network hydrogels typically consist of two networks with distinct mechanical performance. The brittle network acts as a sacrificial component to dissipate mechanical energy, whereas the flexible network maintains overall structural integrity, thereby enhancing both strength and toughness [15]. In contrast, interpenetrating polymer networks emphasize the spatial interweaving of two or more independent polymer networks, with different networks providing distinct functions, such as mechanical support, flexibility, or electrical conductivity [18]. Further regulation of inter-network interactions through chemical, physical, or dynamic mechanisms can promote stress transfer and functional synergy between the networks [19].

The network structure of mechanosensitive hydrogels ultimately governs the conversion of mechanical stimuli into electrical signals. A lower cross-linking density generally promotes polymer-chain mobility and increases deformability, but excessive reduction in network constraints may lead to structural relaxation and signal drift. Conversely, a higher cross-linking density improves dimensional stability and facilitates stress transfer, but may restrict deformation and reduce mechanical compliance. Multinetwork structures can achieve a more favourable balance among mechanical strength, deformability, and fatigue resistance through sacrificial bond dissociation, reversible bond reorganization, and inter-network stress redistribution. These characteristics facilitate the stable detection of long-term, low-amplitude, and periodic thoracoabdominal movements during sleep, as well as airflow-related signals [20].

### 2.2. Sensing Mechanisms

Hydrogel-based mechanical sensors are a class of flexible bioelectronic devices that employ hydrogels as functional sensing materials to convert external mechanical stimuli into measurable electrical signals. Owing to their excellent flexibility, superior biocompatibility, high water content, and suitable mechanical properties comparable with those of biological soft tissues, hydrogels can establish conformal contact with the skin, making them well suited for long-term, continuous sleep respiratory monitoring [21]. During sleep, periodic respiratory movements induce subtle deformations in the chest, abdomen, neck, and nasal regions. When hydrogel-based mechanical sensors attached to these body sites are subjected to mechanical stimuli, such as stretching, compression, bending or pressure, their internal conductive networks undergo structural changes, resulting in variations in electrical parameters, including resistance, capacitance, and voltage [5]. By continuously acquiring and analyzing these electrical signals using appropriate signal-processing algorithms, key respiratory parameters, such as respiratory rate, breathing depth, apnoea events and abnormal breathing patterns, can be monitored in real time. Depending on the signal transduction mechanism, hydrogel-based mechanical sensors for sleep respiratory monitoring can be broadly divided into five types: piezoresistive, capacitive, piezoelectric, triboelectric, and multimodal sensors [4,22].

#### 2.2.1. Piezoresistive Sensors

The piezoresistive sensing mechanism is the most widely adopted and well-established signal transduction mechanism in hydrogel-based flexible sensors for sleep respiratory monitoring. The piezoresistive effect arises when external mechanical stimuli induce deformation of the sensing material, altering the distribution and contact state of the internal conductive network, which in turn leads to variations in electrical resistance [23]. The underlying mechanism depends largely on the type of conductive component incorporated into the hydrogel. For ionically conductive hydrogels, such as those containing mobile ions (e.g., Li^+^, Na^+^, and Ca^2+^), mechanical deformation modifies the migration pathways, concentration distribution, and transport behavior of the ions, thereby altering the ionic conductivity of the hydrogel [16,24]. In contrast, for electronically conductive hydrogels incorporating conductive fillers such as metals, carbon-based materials or MXenes, mechanical deformation changes the relative positions, contact states, and connectivity of the conductive fillers, thereby reconstructing the electron transport pathways and altering the number of conductive channels. These structural changes ultimately produce reversible variations in electrical resistance [19,25,26].

The piezoresistive sensing mechanismof hydrogel-based sensors is commonly evaluated using the relative change in resistance.

(1) $$∆R/R_{0}=\frac{R-R_{0}}{R_{0}}\times 100\%$$

where *R*_0_ is the initial resistance and *R* is the instantaneous resistance measured during deformation.

The strain sensitivity of a piezoresistive sensor is generally quantified by the gauge factor (GF).

(2) $$GF=\frac{R-R_{0}}{R_{0}\varepsilon }$$

where *ε* denotes the applied strain. A higher GF indicates higher sensitivity, meaning that the sensor can generate larger resistance changes under smaller mechanical deformations [27].

Owing to their simple device architecture, high sensitivity, and straightforward signal acquisition, piezoresistive sensors have been widely employed in wearable healthcare devices, motion monitoring, electronic skin, and human–machine interfaces [28]. Nevertheless, hydrogel-based piezoresistive sensors may suffer from signal drift, hysteresis, and reduced long-term repeatability under repeated mechanical loading, which remain important challenges for practical applications [23].

**Figure 1:**
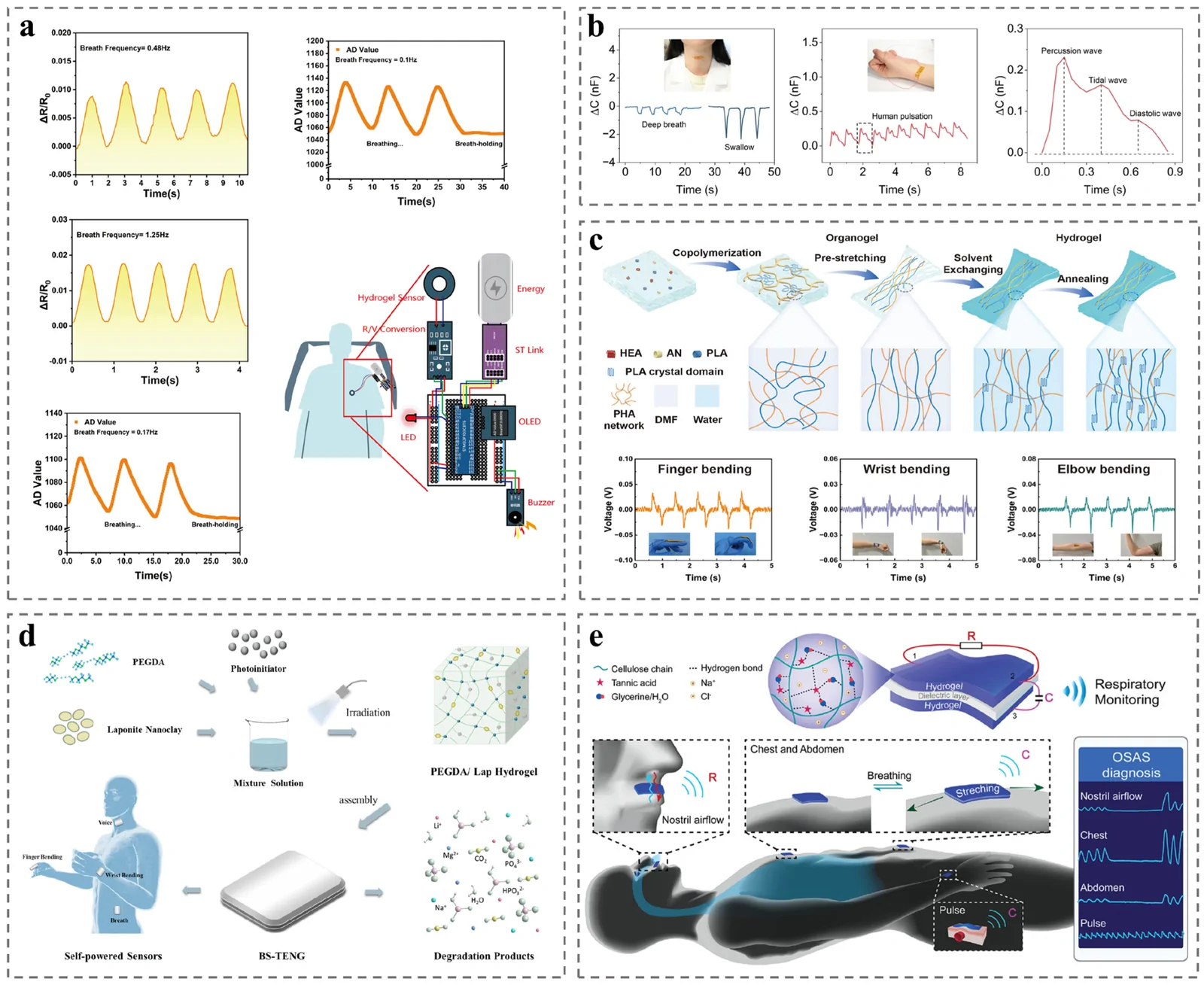
Hydrogel-based mechanical sensors. (**a**) Schematic illustration of a hydrogel-based piezoresistive sensor for respiratory monitoring and its corresponding signal acquisition circuit. Reproduced from reference [19] under Creative Commons license CC-BY-NC-ND, copyright 2026, John Wiley and Sons. (**b**) Hydrogel-based capacitive sensor for real-time monitoring of respiration, swallowing, and pulse. Reproduced from reference [30] under Creative Commons license CC-BY-NC-ND, copyright 2024, Springer Nature. (**c**) Fabrication of piezoelectric PLA hydrogel using a “phase” strategy and its applications in monitoring voltage signals generated during finger, wrist, and elbow movements. Reproduced with permission from reference [31], copyright 2026, John Wiley and Sons. (**d**) Preparation of a multifunctional PEGDA/Lap hydrogel-based BS-TENG and its applications in physiological signal monitoring. Reproduced with permission from reference [32], copyright 2023, American Chemical Society. (**e**) Cellulose-based hydrogel sensor with multimodal sensing capability for respiratory monitoring. Reproduced with permission from reference [21], copyright 2026, John Wiley and Sons.

For example, He and co-workers developed a conductive hydrogel composite consisting of a polyvinyl alcohol (PVA)/silk fibroin (SF) double-network matrix, tannic acid-coated liquid metal, and in situ generated copper nanoparticles. Through multilevel structural regulation, a stable electronically conductive network was established. During periodic respiratory movements, mechanical deformation dynamically reconstructed the internal conductive pathways, thereby leading to reproducible variations in resistance via the piezoresistive effect. The sensor maintained a stable resistive response, with a Δ*R*/*R_0_* fluctuation of less than 5% after 1000 stretching cycles, enabling continuous respiratory monitoring and early detection of apnoea events. These results demonstrate its excellent cyclic stability and considerable potential for long-term sleep respiratory monitoring applications (Figure 1a) [19]. Chen et al. developed a strain sensor based on a biodegradable reduced graphene oxide-silk fibroin hydrogel, achieving high-precision measurements with a tensile strain detection range exceeding 100% and a pressure detection range spanning from 100 Pa to 500 kPa. The sensor’s exceptional performance is attributed to the synergistic optimization of conductive rGO nanosheets with a flexible and biodegradable silk fibroin matrix, which significantly enhances the material’s electrical conductivity and mechanical stability, enabling precise detection of subtle human motion signals (e.g., joint movements of the fingers, wrists, and knees) as well as pulse variations, while its piezoresistive properties facilitate integration into a smart glove-based wearable gesture recognition system, demonstrating substantial potential for applications in human–machine interaction and medical diagnostics [29].

#### 2.2.2. Capacitive Sensors

Capacitive sensors can be utilized to detect mechanical stimuli by measuring the changes in capacitance resulting from variations in the interelectrode distance, effective overlapping area, or dielectric properties. When an external force compresses or displaces one of the electrodes, the distance between the electrodes is altered, leading to a corresponding change in capacitance according to the following equation [33,34].

(3) $$C=\varepsilon_{0}\varepsilon_{r}A/d$$

where *C* is the capacitance, *ε_0_* is the vacuum permittivity, *ε_r_* is the relative permittivity of the dielectric layer, *A* is the effective overlapping area of the two electrodes, and *d* is the interelectrode distance, corresponding to the thickness of the dielectric layer.

Capacitive sensors exhibit several advantages, including low power consumption, rapid response, and excellent signal stability. However, their sensing performance is highly dependent on the design of the dielectric layer and device microstructure, while parasitic capacitance, nonlinear response, and environmental interference may limit their practical applications [35].

He et al. developed a highly conductive and stretchable nanostructured (CSN) ionic gel and fabricated high-performance capacitive sensors using three-dimensional (3D) printing technology. Serving as the dielectric layer, the CSN ionic gel exhibited an ionic conductivity exceeding 3 S m^−1^, stretchability higher than 1500%, ultralow hysteresis (0.4% at a strain of 50%), and remarkable thermal stability over a wide temperature range from −72 to 250 °C. The resulting 3D-printed capacitive sensor enabled real-time monitoring of deep breathing, swallowing, and pulse signals, demonstrating significant potential for wearable healthcare devices and physiological monitoring applications (Figure 1b) [30]. Mo and co-workers developed a capacitive strain sensor based on a dual-crosslinked energy-dissipating hydrogel conductor and a semi-interpenetrating polymer network (semi-IPN) organogel dielectric layer. In this design, a polyacrylamide (PAM)/alginate–Zn^2+^ dual-crosslinked hydrogel served as the flexible conductive electrode, while a poly(acrylic acid)/PVA/borax organogel functioned as the dielectric layer, together forming a stable capacitive sensing architecture. The dynamic dual-crosslinked network effectively dissipated mechanical energy through the reversible breaking and reformation of ionic coordination bonds, thereby imparting excellent stretchability, rapid response, and outstanding cycling durability. The sensor maintained a stable capacitive response under severe mechanical conditions, including repeated stretching, impact loading, and even vehicle rollover. Furthermore, it enabled real-time monitoring of both large-scale body motions, such as wrist, finger, elbow and knee movements, and subtle physiological signals, demonstrating broad application prospects in flexible electronics and wearable health monitoring [36].

#### 2.2.3. Piezoelectric Sensors

The piezoelectric sensing mechanism is based on the generation of electrical charges in response to mechanical deformation. When a piezoelectric material is subjected to external mechanical stress, the relative displacement of positive and negative charge centers induced by internal polarization generates a potential difference across the material, producing a measurable voltage or current without an external power source. Consequently, piezoelectric sensors are regarded as self-powered sensing devices. However, they generally exhibit limited capability for continuous static-pressure detection because electrical signals are generated primarily during dynamic deformation [37]. A flexible piezoelectric hydrogel was developed using a gel-state crystal phase regulation strategy. Through sequential pre-stretching, thermal annealing, and solvent exchange, this approach induced the formation of an oriented β-crystalline phase in polylactic acid, thereby endowing the hydrogel with a stable piezoelectric response. When mounted on the finger, wrist, and elbow, the sensor generated stable, highly reproducible piezoelectric voltage signals during joint flexion, demonstrating excellent capability for human motion monitoring. This work provides a promising strategy for the development of human–machine interfaces, wearable health-monitoring systems, and flexible electronic devices (Figure 1c) [31].

In recent years, advances in ionically conductive hydrogels have led to the emergence of a novel signal transduction mechanism, known as the piezoionic effect, which is fundamentally different from the conventional piezoelectric effect. Under external mechanical stimulation, cations and anions in ionic hydrogels migrate at different rates, resulting in transient charge separation and the generation of an electrical potential. This mechanism provides a new approach to developing self-powered, flexible, wearable sensors [38]. For example, Dobashi et al. systematically investigated the piezoionic behavior of ionic hydrogels and demonstrated that compressive deformation induces ionic concentration gradients owing to the asymmetric migration of cations and anions, thereby generating a measurable electrical signal. Through indentation experiments, the authors further elucidated the microscopic origin of the piezoionic effect. During compression, smaller, more mobile cations migrate more rapidly than larger anions, leading to a transient charge imbalance and the establishment of an internal electric field. These findings provide important theoretical insights into the piezoionic sensing mechanism and support its potential applications in next-generation flexible pressure sensors. The fabricated artificial mechanoreceptor was a poly(acrylic acid-co-acrylamide) hydrogel hemisphere, which was circumscribed by a polyacrylamide hydrogel swollen in 0.1 M KCl on a plane, forming a 4 × 4 touch sensors array. Single- and multi-point touches (approximately 100 g of force) on the sensors array by a finger produced a voltage variation of around −10 mV, which was superimposed on the basic Donnan potential of −50 mV [39].

#### 2.2.4. Triboelectric Sensors

Triboelectric sensors are another important class of self-powered sensors for detecting external mechanical stimuli. Compared with piezoelectric sensors, triboelectric sensors can generate substantially higher output voltages. However, their performance in static pressure sensing is often limited by signal attenuation, rapid charge dissipation, and long-term output instability. Their operating principle relies on the combined effects of contact electrostatic induction and electrification. When two materials with different electron affinities repeatedly come into contact and separate, opposite charges accumulate on their surfaces through triboelectric charge transfer. The resulting electrostatic field drives electron flow through an external circuit, generating alternating voltage or current signals and thereby converting mechanical energy into electrical energy [4,38]. For example, Lu et al. proposed a triboelectric sensor based on an indentation-deformation mechanism. By integrating a microstructured triboelectric layer with a high Young’s modulus and a poly(vinyl alcohol)–phytic acid hydrogel electrode with a low Young’s modulus, the effective contact area was expanded from the conventional cross-sectional interface to the lateral surface, significantly enhancing the triboelectric output. A force–electric coupling model further confirmed that indentation deformation effectively increased the contact area during operation. Consequently, the sensor achieved sensitivities of 7.42 V kPa^−1^ and 1.53 V kPa^−1^ within pressure ranges of 0.248–1.26 kPa and 1.26–25 kPa, respectively, representing improvements of 2.58-fold and 4.94-fold over conventional device architectures. Furthermore, by integrating a bidirectional gated recurrent unit neural network with a bimodal information fusion algorithm, the system achieved a diagnostic accuracy of 98.9% for obstructive sleep apnoea–hypopnoea syndrome, with an average recognition time of only 22.3 ms, demonstrating its considerable potential for intelligent sleep respiratory monitoring [40].

Triboelectric nanogenerators (TENGs) further extend the capabilities of triboelectric sensing by directly harvesting low-frequency mechanical energy from human motion and converting it into electrical energy. This self-powered operating mode makes TENGs particularly attractive for long-term continuous physiological monitoring [41,42]. For instance, Li et al. prepared a PEGDA/Laponite nanocomposite hydrogel using biocompatible PEGDA and Laponite nanosheets, and subsequently fabricated a biodegradable single-electrode triboelectric nanogenerator (BS-TENG). The resulting hydrogel exhibited excellent flexibility, along with enhanced mechanical and electrical properties, including an elongation at break of 1001.8% and a relative change in resistance of 10.8%. The BS-TENG was capable of powering a light-emitting diode without an external power source and simultaneously functioned as an active self-powered sensor for real-time monitoring of respiration and various human movements (Figure 1d) [32]. Similarly, Yang et al. developed a triboelectric nanogenerator based on a liquid metal-composite double-network hydrogel. By improving the dispersion and interfacial stability of the liquid metal within the hydrogel matrix, the device achieved outstanding triboelectric performance, with an open-circuit voltage of 223.32 V, a short-circuit current of 9.78 μA, a transferred charge of 82.33 nC, a maximum power density of 4.30 W m^−2^, and a pressure sensitivity of 0.268 kPa^−1^. The resulting self-powered sensing system enabled real-time respiratory monitoring and, when integrated with machine learning algorithms, also supported speech recognition. These results demonstrate its broad application prospects in sleep respiratory monitoring, wearable electronics, and self-powered healthcare systems [43].

#### 2.2.5. Multimodal Sensors

Although sensors based on a single sensing mechanism offer advantages such as simple device architecture and straightforward signal processing, they are generally limited to detecting a single type of stimulus. Moreover, they are susceptible to environmental interference and motion artifacts, making it challenging to achieve the high accuracy and reliability required for physiological monitoring in complex sleep environments. To address these limitations, multimodal sensing strategies have attracted increasing attention in recent years. By integrating two or more sensing mechanisms within a single device, these sensors enable the simultaneous acquisition of multiple physiological signals, with a focus on mechanical sensing, thereby significantly improving the accuracy and anti-interference capability of sleep respiratory monitoring systems [21,44,45].

For example, a multimodal sensor employing capacitive and resistive sensing modalities can independently detect mechanical deformation and temperature variation through capacitance and resistance outputs, respectively, thereby facilitating the diagnosis of obstructive sleep apnoea syndrome (Figure 1e) [21]. Similarly, Liu et al. studied a hydrogel-electrolyte multimodal sensor based on PVA, sodium alginate, and starch. By simultaneously monitoring mechanical deformation via capacitive signals and temperature changes via resistive signals, the sensor achieved synchronous acquisition of multiple physiological parameters [22].

In another representative study, Ni et al. developed a multimodal sensor based on an MXene/LiCl/polyacrylamide (PAAm)/PVA organohydrogel. By simultaneously generating voltage and resistance signals, the device enabled coordinated monitoring of respiratory airflow and thoracoabdominal movements during sleep, providing more comprehensive respiratory information than conventional single-mode sensors. This multimodal sensing strategy effectively improves the reliability and accuracy of sleep respiratory monitoring and demonstrates considerable potential for next-generation wearable healthcare devices [45].

### 2.3. Composition of Materials

Charge transport in hydrogels can be broadly classified into three modes: ionic conduction, electronic conduction, and mixed ionic–electronic conduction. Ionically conductive hydrogels primarily rely on the migration of mobile ions to facilitate charge transport. Their high flexibility and tissue compatibility make them well suited for physiological signal monitoring [22]. In contrast, hydrogels incorporating conductive components such as metals, carbon-based materials, and MXene, have established electronic transport pathways, with their electrical performance strongly influenced by filler content, dispersion, and conductive network architecture [17,46,47]. Some composite hydrogels involve both ionic and electronic transport behaviours, forming mixed conductive systems that combine flexibility, electrical conductivity, and multimodal sensing capabilities. These distinct transport mechanisms determine how resistance, capacitance, and potential respond to mechanical deformation and thus substantially influence sensing sensitivity, response speed, long-term stability, and sensing modality [45].

#### 2.3.1. Ion-Modified Hydrogels

Following years of rapid development, flexible sensors have gradually evolved from conventional electronically conductive systems to ionically conductive platforms. Owing to their high flexibility, excellent biocompatibility, and tissue-like ionic transport characteristics, ionically conductive sensors have emerged as a major research focus in wearable bioelectronics. Among them, ionically modified hydrogels have attracted particular attention because they construct efficient ionic transport pathways by introducing mobile ions into polymer networks. In addition to enabling efficient and stable mechanoelectrical signal transduction, ion incorporation can significantly enhance the moisture retention, antifreezing capability, self-healing performance, and long-term stability of hydrogels. Consequently, ionically modified hydrogels have been widely explored for long-term continuous healthcare applications, particularly in sleep respiratory monitoring [38,48,49,50].

At present, ion modification is primarily achieved by incorporating inorganic salts, such as LiCl, NaCl, CaCl_2_, and ZnSO_4_, into hydrogel matrices based on PVA, polyacrylamide (PAAm), gelatin, carboxymethyl cellulose (CMC) and sodium alginate. Alternatively, ionic liquids are employed as highly conductive media to establish continuous ionic transport networks within the hydrogel matrix [10,22,24,45,51].

Among these ionic modifiers, LiCl is one of the most widely used because it provides a high concentration of mobile charge carriers, thereby markedly improving the ionic conductivity of hydrogels. Moreover, owing to its high hygroscopicity and ability to regulate the crystallization behavior of water molecules, LiCl substantially enhances water retention, antifreezing performance, and long-term environmental stability [24,52]. Further, LiCl, NaCl, and CaCl_2_ are also extensively employed in ionically conductive hydrogels. NaCl primarily improves ionic conductivity by supplying abundant mobile ions and establishing stable ionic transport pathways. In contrast, CaCl_2_ not only provides mobile ions but also forms a characteristic egg-box ionic cross-linked structure with natural polysaccharides such as sodium alginate, thereby significantly enhancing the mechanical strength, toughness, and cyclic durability of the hydrogel. A representative example is a PVA/SA/starch composite hydrogel, which is first ionically cross-linked with CaCl_2_ to form a robust three-dimensional network and subsequently immersed in an NaCl solution to introduce Na^+^ and Cl^−^ ions, thereby establishing continuous ionic transport pathways. The synergistic effect of Ca^2+^-mediated ionic cross-linking and NaCl-assisted ionic conduction simultaneously improves ionic conductivity, water retention, antifreezing capability, and environmental stability. Such ionically modified hydrogels have been successfully applied in wearable bioelectronic devices for long-term physiological monitoring, including sleep respiratory monitoring [22].

**Figure 2:**
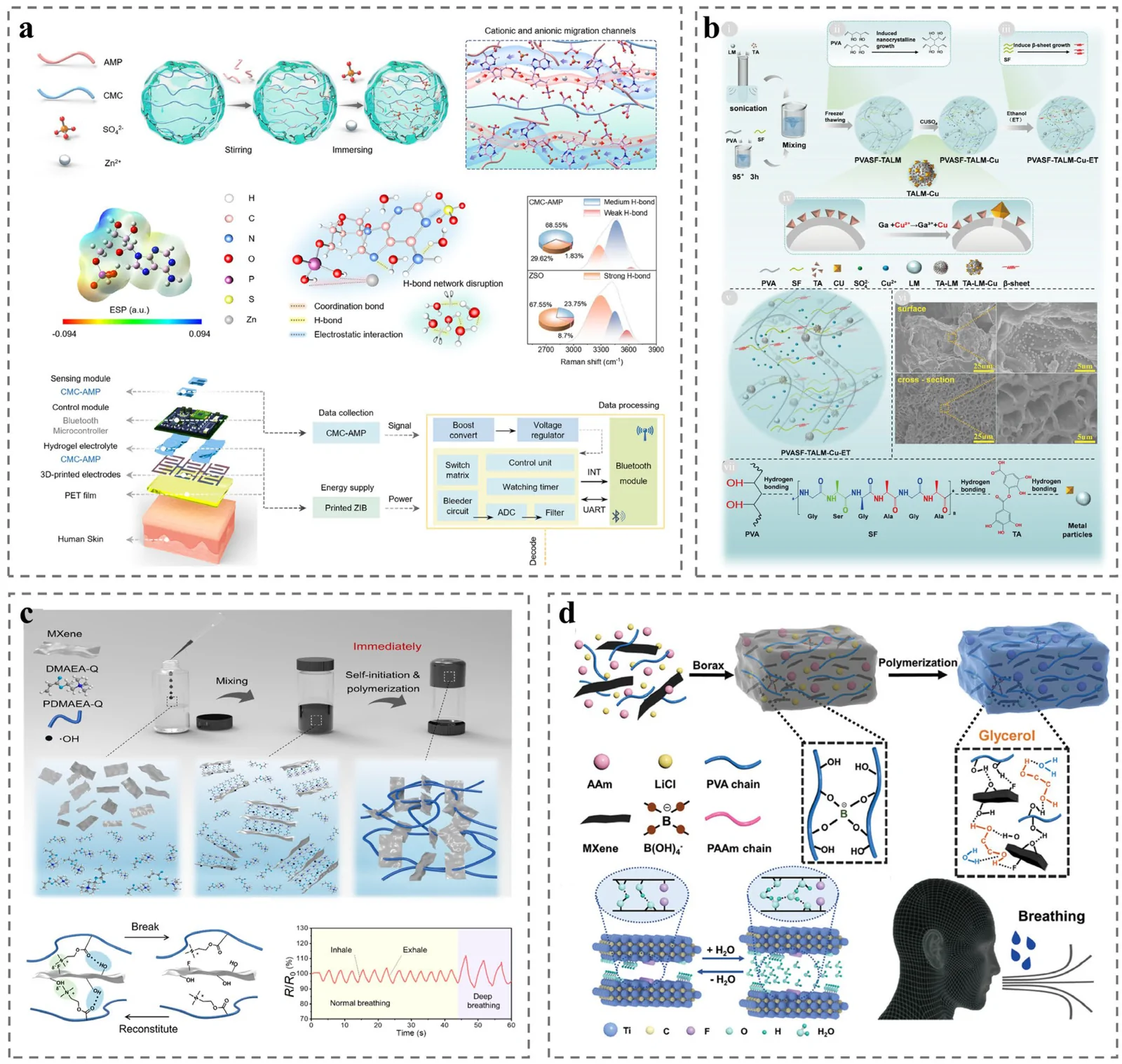
Representative hydrogels for wearable mechanical sensors. (**a**) Schematic illustration of the CMC-AMP hydrogel, the integrated wearable sensing device and the corresponding system architecture. Reproduced with permission from reference [51], copyright 2026, John Wiley and Sons. (**b**) Schematic illustration and microstructure of the PVA/SF−TALM−Cu−ET composite hydrogel. Reproduced from reference [19] under Creative Commons license CC-BY-NC-ND, copyright 2026, John Wiley and Sons. (**c**) Schematic illustration of the MXene-composite hydrogel, its self-healing capability and its applications in respiratory monitoring. Reproduced from reference [47] under Creative Commons license CC-BY, copyright 2023, John Wiley and Sons. (**d**) Applications of the MXene/LiCl/PAAm/PVA organohydrogel for sleep respiratory monitoring. Reproduced with permission from reference [45], copyright 2026, John Wiley and Sons.

Notably, recent ion-modification strategies have evolved beyond simply improving electrical conductivity to integrating sensing and energy-storage functions into a single hydrogel platform. For example, an amphoteric hydrogel based on adenosine monophosphate-modified carboxymethyl cellulose was designed to form biomimetic pseudo-ion channels that facilitate rapid Zn^2+^ transport. When integrated with a flexible zinc-ion microbattery, the hydrogel simultaneously provided a stable power supply and enabled physiological signal acquisition, offering a promising strategy for the development of self-powered wearable bioelectronic systems (Figure 2a) [51].

#### 2.3.2. Metal-Based Hydrogels

Metal-based conductive fillers have been widely incorporated into hydrogels for enhancing their electrical conductivity for wearable biosensing applications. A variety of metallic materials, including transition metals (e.g., iron), noble metals (e.g., silver and gold), liquid metals (e.g., gallium-based alloys), and metal oxides (e.g., zinc- and titanium-based oxides), have been explored for the fabrication of wearable biosensors. In addition to improving electrical conductivity, these metallic components can enhance the selectivity and sensitivity of hydrogel-based mechanical sensors while simultaneously regulating their mechanical properties, magnetic responsiveness, and antibacterial performance [53].

For example, Yan et al. developed a metal-composite hydrogel consisting of a PVA/SF double-network matrix, tannic acid-coated liquid metal microdroplets, and in situ generated copper nanoparticles. The tannic acid coating significantly improved the dispersion stability of the liquid metal, while copper nanoparticles were formed in situ through a galvanic replacement reaction between the liquid metal and Cu^2+^ ions. Subsequently, ethanol treatment induced the β-sheet structures’ formation within the sericin network, thereby synergistically reinforcing both the conductive network and the double-network architecture (Figure 2b). The resulting hydrogel exhibited outstanding overall performance, including a tensile strength of 1.452 MPa, representing an increase of 483% over pristine PVA hydrogel; an elongation at break of 700%; a toughness of 0.483 MJ m^−3^; an electrical conductivity of 0.25 S m^−1^; and excellent antibacterial activity. These properties enabled stable respiratory monitoring and early detection of sleep apnoea events [19].

In another representative study, Wang et al. employed a lignosulfonate (LS)–Fe^3+^ dynamic redox system to rapidly initiate polymerization at room temperature and to construct a hierarchically dynamic cross-linked network through Fe^3+^–polymer coordination interactions, boronate ester bonds, and hydrogen bonding. The resulting nanocomposite hydrogel exhibited a wide sensing range (500%), a rapid response of 139 ms, and a high gauge factor (GF = 8.98). The sensor enabled accurate speech recognition and the detection of subtle human motions, demonstrating considerable potential for sleep management and real-time health monitoring, particularly for elderly individuals [17].

#### 2.3.3. Carbon-Based Hydrogels

Carbon-based materials have been widely incorporated into hydrogels owing to their large specific surface area, excellent electrical conductivity, superior chemical stability, and outstanding mechanical properties [54]. By introducing conductive carbon nanomaterials, such as carbon nanotubes, graphene, rGO, carbon fibers (CFs), and carbon black, into polymer networks, continuous electron-transport pathways can be established throughout the hydrogel matrix. Consequently, carbon-based composite hydrogels exhibit excellent mechanical strength, outstanding cyclic stability, and high electrical conductivity, making them highly attractive for wearable biosensing applications [27,46,55,56].

Meng et al. developed a skin–hair-inspired sensor based on an alkali-treated polyacrylonitrile/ionic liquid/electrostatically flocked carbon fiber (aPAN/IL/CF) nanofibrous hydrogel. This sensor demonstrated exceptionally high sensitivity for both airflow and strain sensing, exhibiting an airflow response time of only 0.10 s, a minimum detectable airflow velocity of 0.033 m s^−1^, a tensile response time of 0.6 s and a maximum GF of 76.1. Furthermore, the lightweight, flexible, and self-adhesive characteristics of the aPAN/IL/CF nanofibrous hydrogel enabled conformal integration with wearable medical devices for continuous monitoring of physiological signals associated with obstructive sleep apnoea, including oronasal airflow, thoracoabdominal respiratory movements and pulse signals. Beyond respiratory monitoring, the sensor also demonstrated potential for gesture recognition and assistive communication in patients with speech impairments [27]. In another representative study, Kim et al. developed a stretchable graphene–hydrogel bioelectronic interface by integrating a conductive graphene layer with a compliant hydrogel substrate. This hybrid structure provided excellent mechanical compatibility with the skin and soft tissues while maintaining high electrical conductivity. The resulting device exhibited outstanding stretchability and stable electrical performance, enabling long-term conformal attachment to the human body for continuous physiological signal acquisition. During respiratory monitoring, the sensor accurately detected subtle chest and abdominal deformations induced by breathing, enabling reliable discrimination among different respiratory states, and continuous, real-time monitoring of respiratory rate [46].

#### 2.3.4. MXene-Based Hydrogels

MXenes are a family of two-dimensional transition metal nitrides, carbides, and carbonitrides that are typically synthesized by selectively etching the A-layer from MAX-phase precursors [57,58]. When incorporated into hydrogel matrices, MXene nanosheets establish continuous, stable electron-conducting networks throughout the polymer framework, thereby markedly enhancing the electrical conductivity, sensing sensitivity, and response speed of hydrogel-based mechanical sensors. Moreover, surface functional groups on MXene, including –OH, –O, and –F, can form hydrogen bonds and other interfacial interactions with polymer chains, thereby further improving the mechanical strength, water retention capability, and environmental stability of hydrogels while preserving their flexibility and biocompatibility [59,60].

Huang et al. developed a multifunctional MXene-based nanocomposite hydrogel that combines facile fabrication process with ultrahigh stretchability (>5000%) and excellent three-dimensional printability. The hydrogel also exhibited outstanding mechanical and electrical self-healing capabilities. As a wearable respiratory sensor, it enabled real-time monitoring of both respiratory rate and breathing depth. Different breathing patterns, including normal and deep breathing, produced distinct variations in resistance in response to changes in nasal airflow temperature and pressure, enabling accurate identification of respiratory states (Figure 2c) [47]. In addition, the synergistic combination of MXene and LiCl enabled the construction of an ion–electron hybrid conductive network within a PVA/polyacrylamide (PAAm) double-network hydrogel, providing the sensor with excellent mechanical robustness and high humidity sensitivity. The resulting humidity sensor exhibited rapid response over a relative humidity range of 40–85% and achieved a sensitivity of −103.4% RH^−1^. It also demonstrated an ultrahigh elongation at break exceeding 3000% together with excellent environmental adaptability. These superior properties were attributed to the synergistic effects of the chemically cross-linked double-network architecture and the multiple hydrogen-bonding interactions among glycerol, water molecules, and polymer chains. A wearable monitoring system based on this hydrogel-based mechanical sensor enabled real-time detection of humidity variations during respiration and continuous monitoring of respiratory behavior during sleep, demonstrating its considerable potential for long-term respiratory monitoring during sleep (Figure 2d) [45].

### 2.4. Fabrication Strategies

#### 2.4.1. Hydrogel Matrix Fabrication

**In situ polymerization** is one of the most widely employed strategies for fabricating conductive hydrogels. In this approach, monomers, cross-linking agents, initiators, and other functional components are first uniformly dispersed to form a homogeneous precursor solution. Polymerization is then initiated directly by thermal, photochemical, or redox activation, leading to the formation of a three-dimensional cross-linked hydrogel network in situ [61,62,63]. Thermal polymerization typically employs persulphate initiators, such as ammonium persulphate (APS) or potassium persulphate, which decompose under heating conditions to generate free radicals that initiate the polymerization of vinyl monomers, such as acrylamide and acrylic acid [64]. In contrast, photo-initiated polymerization employs photoinitiators that generate free radicals under ultraviolet irradiation, offering rapid polymerization kinetics, and excellent spatial and temporal control over the polymerization process [62]. Redox-initiated systems typically employ an initiator system comprising persulphates and a reducing agent, such as APS/N,N,N′,N′-tetramethylethylenediamine, which can rapidly generate free radicals and complete polymerization at lower temperature or room temperature, making them suitable for temperature-sensitive systems [61].

Owing to its simple processing procedure, broad material compatibility, homogeneous distribution of functional components and excellent scalability, in situ polymerization has become the predominant method for constructing hydrogel matrices in hydrogel-based mechanical sensors for sleep respiratory monitoring. Nevertheless, once the polymer network is formed, its microstructure is difficult to be regulated. The cross-linking density, pore architecture, and mechanical properties are strongly influenced by the monomer composition, cross-linker concentration, and polymerization conditions. Consequently, further improvements in the overall performance of hydrogel-based mechanical sensors often require incorporating dynamic cross-linking strategies, double-network architectures, or other multifunctional network designs [5].

**Chemical cross-linking** is a widely used strategy for constructing hydrogel networks by forming stable chemical bonds between polymer chains through cross-linking agents or chemical reactions. This approach imparts excellent structural integrity, mechanical strength, and long-term stability to hydrogels. Moreover, by tailoring the type of cross-linking chemistry, cross-linking density, and network architecture, the elasticity, toughness, water content, and electrical conductivity of hydrogels can be effectively regulated, thereby providing a robust matrix for flexible bioelectronic devices [17]. Despite these advantages, conventional chemical cross-linking is generally based on permanent covalent bonds, which limit the dynamic adaptability of hydrogel networks. In addition, some commonly used cross-linking agents may exhibit cytotoxicity, and the permanently cross-linked network cannot be readily reconfigured after gelation, restricting the realization of dynamic functions such as self-healing, self-adaptation, and stress relaxation. Consequently, recent research has increasingly shifted from conventional permanent covalent cross-linking towards dynamic covalent chemistry and multiple cross-linking strategies that integrate different types of reversible interactions. Based on the nature of the cross-linking interactions, chemical cross-linking strategies can generally be classified into permanent covalent and dynamic chemical cross-linking. Permanent covalent cross-linking commonly employs cross-linkers such as N,N′-methylenebisacrylamide and glutaraldehyde to establish stable covalent linkages between polymer chains, thereby significantly improving the mechanical strength, structural integrity, and dimensional stability of hydrogels [16].

In contrast, dynamic chemical cross-linking relies on reversible interactions, including dynamic covalent bonds (e.g., boronate ester bonds and Schiff base bonds) and metal coordination bonds. These dynamic bonds can undergo reversible dissociation and reformation in response to external stimuli, enabling hydrogels to autonomously repair structural damage, adapt to repeated mechanical deformation, and resist fatigue accumulation [17]. For example, the dynamic boronate ester bonds formed between borax and PVA impart excellent self-healing capability while maintaining the mechanical integrity of the hydrogel during repeated deformation [65].

**Freeze–thaw cycling** is a widely used physical cross-linking strategy for hydrogel fabrication that eliminates the need for chemical cross-linking agents. It is particularly suitable for semicrystalline polymers, such as PVA. During repeated freezing and thawing cycles, the polymer chains become locally concentrated as water crystallizes, promoting the formation of microcrystalline domains. These domains act as physical cross-linking junctions, giving rise to a stable three-dimensional network that imparts excellent mechanical properties, flexibility, and biocompatibility to the hydrogel [66].

Freeze–thaw processing is simple, environmentally friendly and easy to implement, and the resulting hydrogels generally exhibit high transparency, excellent flexibility, and high biocompatibility. Consequently, this technique has been widely adopted for the fabrication of wearable bioelectronic devices. However, because the hydrogel network is stabilized solely by physical cross-linking, freeze–thaw hydrogels often exhibit limited mechanical strength, structural stability, and functional tunability compared with chemically cross-linked systems. These limitations may restrict their performance in demanding applications requiring prolonged mechanical deformation and long-term operation. To overcome these shortcomings, recent studies have increasingly combined freeze–thaw cycling with complementary strategies, including chemical cross-linking, ionic cross-linking, and the incorporation of conductive fillers, to construct multifunctional hydrogel networks. These hybrid approaches effectively enhance the mechanical robustness, electrical conductivity, environmental stability, and durability of hydrogels, thereby meeting the performance requirements of long-term wearable sensing applications, particularly sleep respiratory monitoring [16].

#### 2.4.2. Fabrication of Hydrogel-Based Composite Structures

**Ion immersion** is a simple and effective post-treatment strategy for fabricating ionically conductive hydrogels. In this method, a preformed hydrogel is immersed in an ionic salt solution, allowing mobile ions to diffuse into the polymer network and establish continuous ionic transport pathways without disrupting the original hydrogel architecture. In addition to enhancing ionic conductivity, ion immersion can reinforce the polymer network and improve the environmental stability of hydrogels. Because this strategy does not require reconstruction of the hydrogel matrix, it is facile to implement and can be performed under mild conditions. Furthermore, the electrical conductivity, water retention capability, and swelling resistance of the hydrogel can be readily tailored by adjusting the ion species, ion concentration, and immersion time [67,68,69].

**Figure 3:**
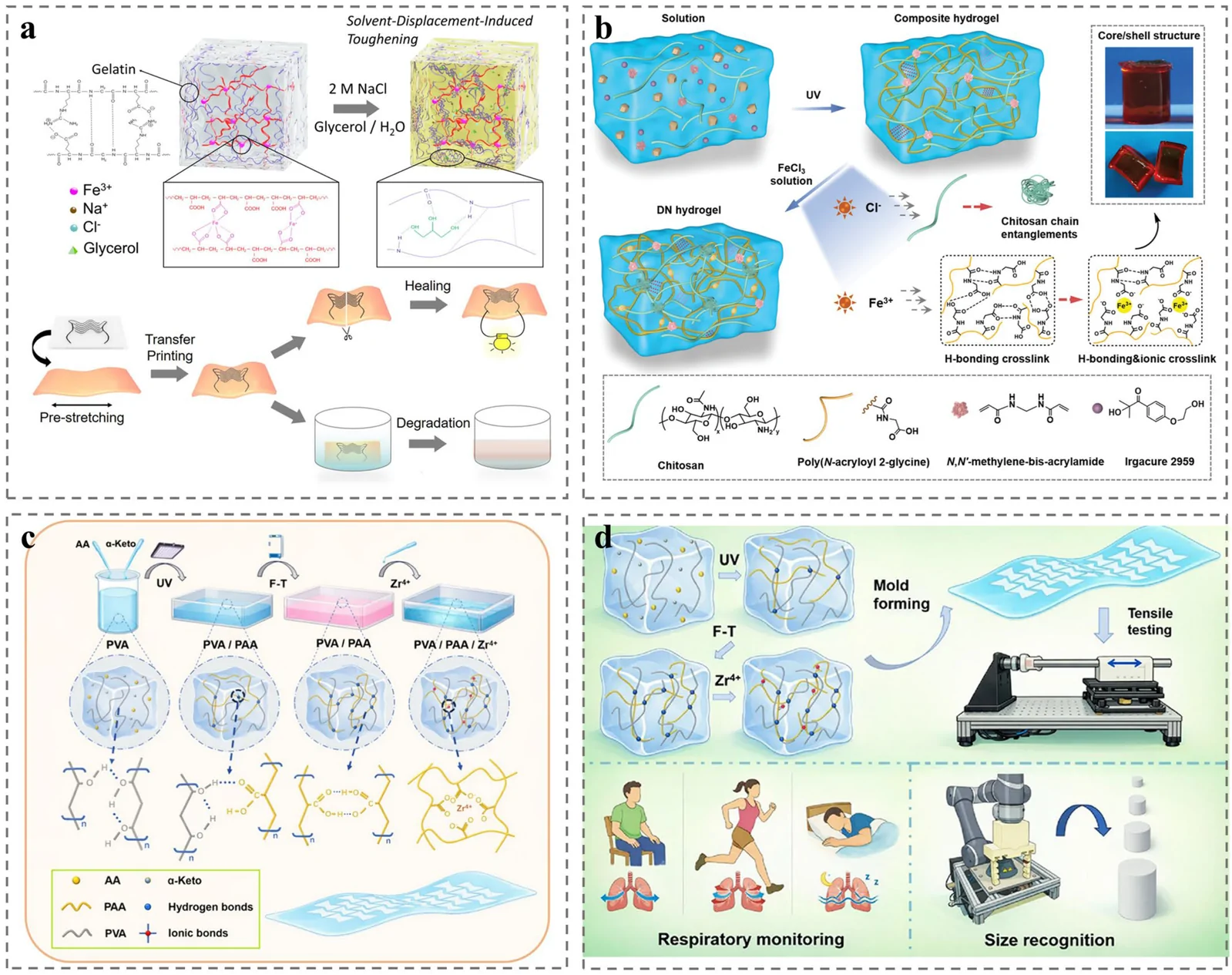
Fabrication strategies and structural regulation of hydrogel-based mechanical sensors. (**a**) Schematic illustration of the preparation of a gelatin/poly(acrylic acid)/glycerol organohydrogel sensor. Reproduced with permission from reference [69], copyright 2020, American Chemical Society. (**b**) Schematic illustration of the CS/PACG double-network (DN) hydrogel with a shell−core structure, including the one-step immersion process for regulating the CS/PACG dual network and the multiple interactions between polymer chains and various ions. Reproduced from reference [70] under Creative Commons license CC-BY, copyright 2023, John Wiley and Sons. (**c**) Schematic illustration of the fabrication process and cross-linking mechanism of the PVA/PAA/Zr^4+^ hydrogel sensor. Reproduced with permission from reference [62], copyright 2026, American Chemical Society. (**d**) Applications of the PVA/PAA/Zr^4+^ hydrogel sensor for respiratory analysis and size recognition. Reproduced with permission from reference [62], copyright 2026, American Chemical Society.

For example, Di et al. first prepared physically cross-linked PVA hydrogels through repeated freeze–thaw cycles and subsequently immersed them in a saturated NaCl solution. The diffusion of Na^+^ and Cl^−^ ions throughout the polymer network increased the effective network density, significantly enhancing both the mechanical properties and ionic conductivity of the hydrogel. The resulting PVA–NaCl hydrogel exhibited a tensile strength of 8.03 MPa, a toughness of 28.7 MJ m^−3^, and an ionic conductivity of 7.14 S m^−1^. As a strain sensor, it operated reliably over a strain range of 0.2–400% with a GF of 0.989, enabling accurate monitoring of physiological signals, including respiration and joint motion [68]. Similarly, Fang et al. developed a gelatin/poly(acrylic acid) (Gel/PAA) dual-dynamic network hydrogel and subsequently treated it by immersion in a NaCl/glycerol–water solution to further reinforce the gelatin network. The resulting hydrogel exhibited a toughness of 1.34 MJ m^−3^ and an ionic conductivity exceeding 7 mS cm^−1^. It maintained stable electrical conductivity under tensile strains of up to 300% and retained its sensing performance after repeated deformation. In addition, the hydrogel demonstrated excellent resistance to dehydration for longer than six months, together with self-healing capability and rapid biodegradability under ambient conditions (Figure 3a) [69].

In another representative study, Dou et al. proposed a one-step ion-immersion strategy to fabricate hydrogels with gradient composite structures through metal-ion diffusion. A chitosan (CS)/poly(N-acryloyl-2-glycine) (PACG) double-network hydrogel was first prepared by ultraviolet (UV)-initiated polymerization and subsequently immersed in an FeCl_3_ solution. During immersion, Fe^3+^ and Cl^−^ ions gradually diffused into the hydrogel matrix. Fe^3+^ ions coordinated with the carboxylate (–COO^−^) groups of PACG to establish an additional ionic cross-linked network, while Cl^−^ ions promoted contraction and entanglement of the chitosan chains. As a result, a highly cross-linked, rigid outer layer formed, while the interior retained a compliant hydrogel structure, producing a characteristic soft-core/stiff-shell architecture. The resulting hydrogel exhibited exceptional mechanical robustness and swelling resistance, with a compressive modulus ranging from 0.17 to 4.25 MPa and a swelling ratio spanning from −45% to 44,000%. Furthermore, by varying the immersion cycles and metal-ion species, the hydrogel exhibited reversible transitions between swelling states, providing an effective strategy for engineering mechanically robust hydrogel structures (Figure 3b) [70].

**In situ compositing** is an effective fabrication strategy in which functional nanomaterials or conductive components are incorporated during hydrogel network formation. This approach enables the uniform distribution of functional fillers throughout the polymer matrix and promotes strong interfacial interactions between the incorporated materials and the hydrogel network, thereby overcoming the limitations of conventional physical blending, such as poor dispersion and weak interfacial adhesion [71]. Currently, in situ compositing has been widely employed for integrating liquid metals, MXenes, carbon nanomaterials, and nanoparticles into hydrogels, providing an important materials platform for the development of high-performance wearable bioelectronic devices and flexible sensors, including sleep respiratory monitoring systems [72,73,74].

Liu et al. developed a multifunctional hydrogel patch for sleep monitoring using an in situ composite strategy. In this system, poly(acrylic acid) was used as the hydrogel matrix, and conductive nanomaterials, including MXene and carbon nanotubes, were incorporated simultaneously during hydrogel network formation. The introduced nanomaterials were uniformly distributed within the polymer framework and established continuous electron transport pathways, thereby providing the composite hydrogel with enhanced mechanical sensitivity and stable electrical signal output [72]. Similarly, Yu et al. fabricated an ionically conductive PVA/PAA/Zr^4+^ hydrogel-based electronic skin via an in situ compositing approach. In this system, a PVA/PAA polymer network was initially constructed through one-step in situ photopolymerization, followed by freeze–thaw-induced crystallization of PVA and physical cross-linking. Subsequently, Zr^4+^ ions were introduced to coordinate with carboxyl groups in the PAA chains, forming a dynamic ionic cross-linked network. Through the in situ incorporation process, Zr^4+^ ions were uniformly distributed throughout the hydrogel matrix and synergistically interacted with the dense hydrogen-bond network, resulting in a multifunctional cross-linked architecture with enhanced mechanical robustness and improved stability of conductive pathways (Figure 3c). Furthermore, the PVA/PAA/Zr^4+^ composite network could effectively dissipate mechanical energy through reversible bond dissociation and reconstruction, thereby improving fatigue resistance and long-term sensing stability. The resulting ionically conductive hydrogel exhibited excellent mechanical performance, including a maximum tensile stress of 552.9 kPa and an elongation at break of 629.4%. It also demonstrated a broad strain sensing range of 0.1–200%, a strain resolution of 0.1%, and a rapid response of 94.2 ms. The electronic skin fabricated from this composite hydrogel exhibited conformal adhesion to the abdominal skin and enabled continuous monitoring of various respiratory patterns, highlighting its potential for wearable sleep respiratory monitoring applications (Figure 3d) [62].

**Electroflocking** is a fiber-alignment technique that uses an external electric field to induce the directional arrangement of conductive fibers, followed by their integration into a hydrogel matrix to construct ordered conductive sensing networks. Compared with conventional composites containing randomly distributed fillers, electroflocking enables high-aspect-ratio fibers to align with the electric field, generating continuous and controllable conductive pathways. This ordered architecture improves electron transport efficiency, mechanical signal transduction capability, and sensing stability [75,76]. For example, researchers developed a biomimetic fiber-array hydrogel-based mechanical sensor using an electrostatic flocking strategy. In this design, an alkali-treated polyacrylonitrile/ionic liquid nanofibrous hydrogel served as the substrate, while CFs were vertically deposited onto the hydrogel surface under an electrostatic field, forming an ordered fiber array inspired by the hierarchical structure of human hair. The vertically aligned carbon fibers served as electron transport channels, and their contact–separation behavior under external mechanical deformation regulated the conductive pathways, resulting in measurable variations in resistance. Meanwhile, the ionic liquid-containing aPAN/IL hydrogel matrix provided a continuous ionic transport network, thereby improving signal transmission efficiency, and enhancing the sensor’s adhesion to both skin and medical catheters. By integrating electronic and ionic conduction mechanisms, this biomimetic composite structure enabled real-time monitoring of multiple physiological signals, including oral and nasal respiration, abdominal respiratory movements, and sleep pulse, demonstrating potential for auxiliary diagnosis of sleep apnoea syndrome [27].

Furthermore, high-density fiber arrays fabricated through electrostatic flocking can exploit the large aspect ratio and highly oriented arrangement of fibers to enhance responses to subtle mechanical disturbances and airflow variations [77]. For instance, Luo et al. developed an ultrasensitive fluffy airflow sensor by uniformly and firmly embedding CFs into the surface of a PVA fiber substrate. The resulting fibrous architecture exhibited outstanding airflow-sensing performance, including an ultrafast response of 0.103 s, a low airflow detection limit of 0.068 ms^−1^, a broad detection range of 0.068–16 ms^−1^, and consistent multidirectional airflow responses. The sensor was capable of accurately distinguishing acoustic signals from respiratory airflow and detecting human and object motion under different postures and velocities, highlighting its potential for wearable respiratory monitoring and intelligent sensing applications [75].

## 3. Application Challenges and Optimization Strategies

Hydrogel-based mechanical sensors have undergone rapid development in recent years for applications including respiratory signal acquisition, sleep-state monitoring, and sleep apnoea detection, benefiting from advances in conductive network construction, functional material integration, and structural engineering. However, the practical applications of these sensors for sleep monitoring remain challenging due to several factors, including the low-amplitude and prolonged nature of respiratory signals, involuntary body movements during sleep, dynamic changes in the warm, humid skin environment, and the requirement for continuous, comfortable long-term wear. These challenges primarily involve signal accuracy, response speed, long-term stability, and wearing comfort.

To provide a comprehensive comparison of the performance characteristics of different hydrogel-based mechanical sensors, Table 1 summarizes representative hydrogel-based mechanical sensors reported in recent years, covering their material compositions, fabrication strategies, sensing mechanisms, key performance parameters, and practical application scenarios.

### 3.1. High Accuracy

Accurate detection of weak, dynamic, and continuously varying physiological signals is essential for the applications of hydrogel-based mechanical sensors in sleep apnoea monitoring. However, in real-world sleep environments, respiratory signals are typically characterized by low-amplitude, low-frequency periodic mechanical deformations. These subtle signals are highly susceptible to interference from body movements, changes in sleeping posture and variations in the sensor–skin interface, resulting in signal distortion, false event detection, and reduced monitoring accuracy [72,78]. Consequently, sensor’s accuracy depends not only on the intrinsic sensitivity of the sensing material, but also on signal resolution, repeatability, resistance to motion artefacts, and the consistency of respiratory event detection. For sleep respiratory monitoring, the accuracy of a sensor can be evaluated by comparing its measurements of respiratory rate, respiratory amplitude, and apnoea events with those obtained using established reference methods.

During sleep, the mechanical deformations associated with thoracic and abdominal movements, as well as nasal airflow, are relatively small in magnitude, placing stringent demands on sensor sensitivity and strain resolution. To address this challenge, dual-network hydrogels, dynamically cross-linked hydrogels, and nanomaterial-reinforced hydrogels have been extensively investigated. These materials improve the efficiency of mechanical signal transmission through synergistic interactions among multiple network structures, enabling more precise detection of respiratory rhythms and subtle amplitude variations [19,45,50]. For example, by simultaneously incorporating phytic acid and hydrophobic lauryl methacrylate (LMA) into a PAM/CMC network, researchers constructed a multidynamic cross-linked hydrogel based on hydrogen bonding, ionic interactions, and hydrophobic associations. This synergistic network not only exhibited excellent mechanical strength, with a tensile strength of 1.06 MPa, but also achieved a high ionic conductivity of 1.45 S m^−1^, enabling reliable detection of sleep-related respiratory abnormalities in patients with heart failure [50].

**Figure 4:**
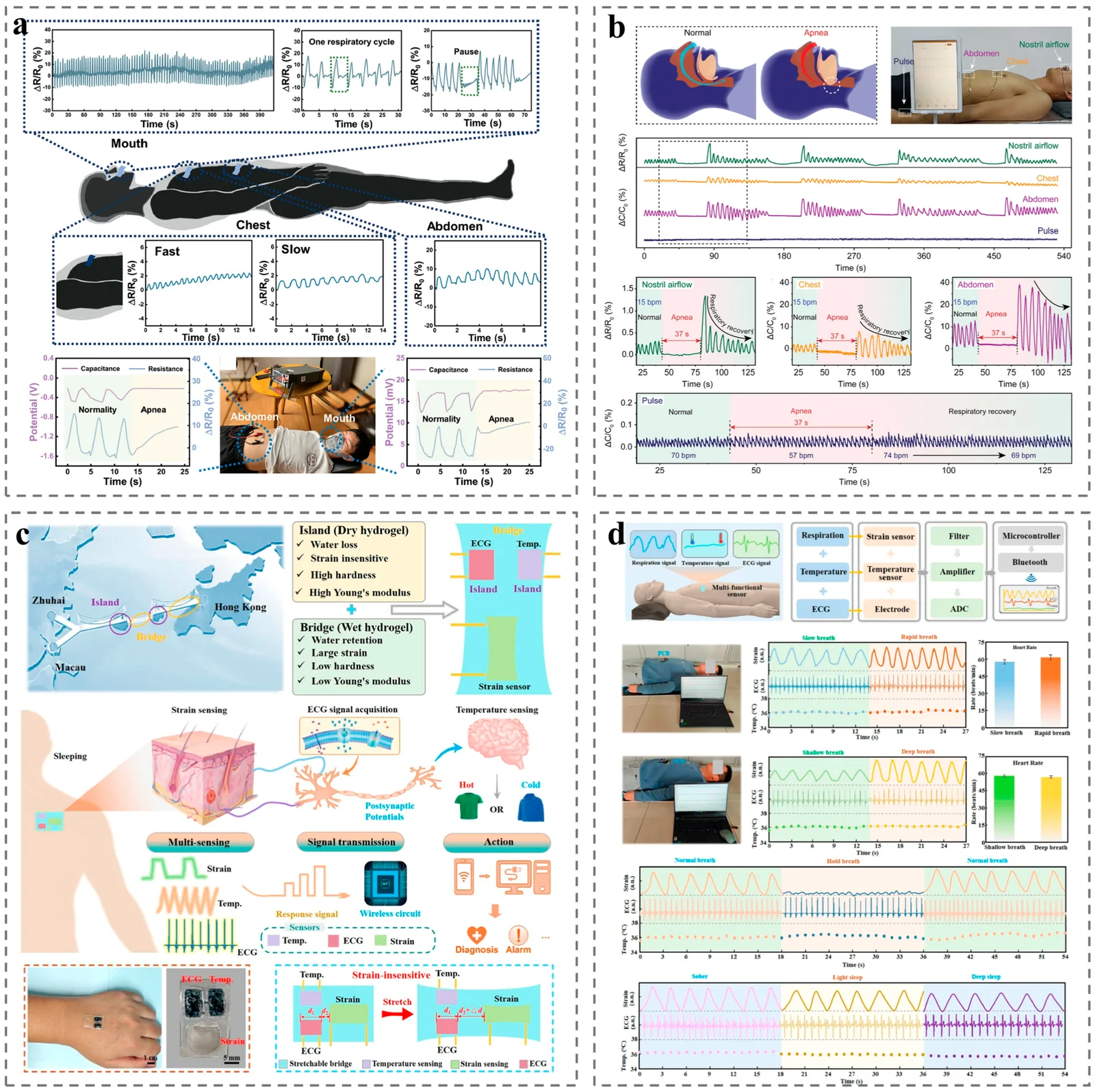
Strategies for improving the accuracy of sleep-breathing monitoring using hydrogel-based mechanical sensors. (**a**) Multimodal MXene/LiCl/PAAm/PVA hydrogel sensor for obstructive sleep apnoea monitoring. Reproduced with permission from reference [45], copyright 2026, John Wiley and Sons. (**b**) The CH-GT100 multimodal hydrogel sensor improves sleep apnoea detection accuracy by cross-validating signals acquired from multiple sites and sensing modalities. Reproduced with permission from reference [21], copyright 2026, John Wiley and Sons. (**c**) Schematic illustration of a multifunctional hydrogel patch with an island−bridge structure. Reproduced with permission from reference [72], copyright 2026, American Chemical Society. (**d**) Simultaneous monitoring of respiratory activity, electrocardiogram (ECG) and body temperature using the multifunctional hydrogel patch. Reproduced with permission from reference [72], copyright 2026, American Chemical Society.

Another major challenge arises from motion artifacts generated by non-respiratory activities during sleep. Body turning, involuntary limb movements, and muscle contractions produce mechanical stimuli that can overlap with respiratory signals, leading to inaccurate identification of respiratory events. Multimodal hydrogel-based mechanical sensors provide an effective solution by simultaneously acquiring multiple physiological signals with different sensing modalities [21,45]. For instance, Ni et al. developed a multimodal sensor based on an MXene/LiCl/PAAm/PVA organohydrogel that simultaneously outputs resistance and voltage signals, enabling independent monitoring of thoracic and abdominal movements, as well as humidity changes in exhaled airflow. During apnoeic events, both the oral airflow and abdominal motion exhibited nearly flat response curves. Cross-validation of signals acquired from different sensing modalities and locations effectively reduced false classifications associated with single-signal monitoring, thereby improving the diagnostic accuracy for sleep-related breathing disorders (Figure 4a) [45]. Similarly, the CH-GT100 multimodal hydrogel-based mechanical sensor simultaneously monitors nasal airflow, thoracic and abdominal movements, and pulse signals. By integrating information from multiple sensing sites and signal types, the device accurately identifies apnoeic events, substantially reducing false detections associated with single-sensor systems and improving the accuracy of obstructive sleep apnoea diagnosis (Figure 4b) [21].

The skin surface is characterized by softness, dynamic deformation and sweat secretion, as a result, conventional sensors are prone to issues such as interface slippage, changes in contact impedance and baseline drift. Self-adhering hydrogels, tissue-matching flexible networks, and fatigue-resistant structures can improve stable contact between the sensor and the skin [72]. Liu et al. designed a multifunctional hydrogel patch based on an island-bridge structure. Through the differentiated design of “islands” made of low-water-content hydrogel and “bridges” made of high-water-content hydrogel, effective decoupling of the strain and electrode regions was achieved, enabling the sensor to maintain stable signal output even during movement (Figure 4c). This patch can simultaneously monitor respiratory, electrocardiogram, and body temperature signals; the different physiological parameters can cross-validate one another, effectively reducing misinterpretations caused by motion artefacts affecting individual signals, and improving the accuracy and reliability of multi-parameter monitoring during sleep (Figure 4d) [72].

With the development of smart technologies, artificial intelligence algorithms are gradually being applied to the data analysis process of hydrogel-based mechanical sensors. Due to the complexity and individual variability of sleep respiration signals, high-precision recognition is difficult to achieve relying solely on traditional threshold-based judgement. By combining machine learning and deep learning algorithms, feature extraction and pattern recognition can be performed on large volumes of physiological signals, enabling the automatic classification of apnoea, hypopnea, and abnormal breathing patterns, thereby further enhancing the accuracy and intelligence of sleep monitoring systems [40].

### 3.2. Rapid Response

Sleep respiration monitoring requires sensors with rapid response to accurately capture weak, continuously changing respiratory signals in real time. When the sensor response is insufficiently fast, signal delay and waveform distortion may occur, compromising the continuous recording of respiratory cycles and potentially leading to missed detection of transient abnormal events, such as brief apnoea episodes. Therefore, improving the dynamic response capability of hydrogel-based mechanical sensors is essential for achieving high-precision, real-time sleep monitoring. Rapid response is typically evaluated in terms of response and recovery time. However, the stability of these response characteristics under repeated cyclic loading, as well as the temporal synchronisation between the sensor output and the actual respiratory cycle, should also be considered. For sleep respiratory monitoring, the response speed should be sufficient for the real-time acquisition of continuous, low-frequency respiratory signals, rather than being optimized solely for an extremely short response time. In recent years, researchers have mainly focused on improving sensor’s response speed through the optimization of hydrogel network architectures, construction of efficient conductive pathways, and development of microstructured sensing interfaces [21,26,40]. For example, the CH-GT hydrogel sensor achieves an ultrafast response of approximately 20 ms by constructing a highly resilient dual-network structure. This sensor can reliably detect subtle mechanical signals, including impacts from 10 mg microdroplets, heartbeat-induced deformations, blinking, and body movements, demonstrating excellent dynamic sensing performance. It has also been successfully applied for the diagnosis of obstructive sleep apnoea syndrome [21].

Beyond improving the intrinsic response characteristics of hydrogel materials, intelligent signal-processing algorithms play an increasingly crucial role in enhancing the real-time performance of sleep monitoring systems. By integrating respiratory and pulse signals and employing a bidirectional gated recurrent unit network for real-time classification, the temporal characteristics and complementary information of multiple physiological signals can be effectively exploited. This approach achieved a diagnostic accuracy of 98.9% with a recognition time of only 22.3 ms, demonstrating the potential of combining multimodal sensing with artificial intelligence for rapid respiratory event identification [40]. In the future, the integration of highly responsive hydrogels, microstructural engineering, and artificial intelligence algorithms is expected to enable next-generation sleep apnoea monitoring systems with rapid signal acquisition, real-time analysis, and intelligent early-warning capabilities.

### 3.3. Long-Term Stability

Sleep breathing monitoring generally requires continuous operation over several hours or even multiple nights. Therefore, hydrogel-based mechanical sensors should not only maintain stable mechanical properties but also preserve structural integrity and consistent sensing performance during prolonged use. However, owing to their high water content, conventional hydrogels are susceptible to water evaporation, swelling, and network fatigue during long-term operation. These issues can cause deterioration in mechanical properties, dimensional instability, and signal drift, ultimately reducing the reliability of monitoring outcomes [16,45,50].

Dehydration of hydrogels is a major factor limiting their long-term wearability and sensing stability. Prolonged exposure to air promotes the continuous evaporation of free water from the hydrogel network, leading to the mass loss and network shrinkage. Water loss can also reduce ionic conductivity and elasticity, resulting in decreased sensitivity and baseline drift [79]. Therefore, the dehydration resistance of hydrogels for sleep respiratory monitoring should be evaluated under conditions that reflect long-term use. In addition to short-term water loss rates, the changes in moisture retention, mechanical performance, and sensing stability should be assessed during continuous monitoring over 6–10 h and repeated use across multiple nights. Reducing the proportion of free water and strengthening interactions between water molecules and the polymer network are effective strategies for improving the dehydration resistance of hydrogels [80].

Currently, strategies for improving the dehydration resistance of hydrogels mainly include surface encapsulation, solvent engineering, and ionic incorporation. Surface encapsulation typically involves applying a flexible barrier layer with hydrophobic properties or low water-vapour permeability to the hydrogel surface, thereby reducing water diffusion and evaporation into the surrounding environment. However, the barrier properties and flexibility of the encapsulating layer should be carefully balanced. An excessively thick or rigid layer may restrict hydrogel deformability and compromise conformal adhesion to the skin [81]. Solvent engineering improves dehydration resistance by regulating the state of water and its interactions with the polymer networks. A higher cross-linking density and smaller pore size of network can restrict the migration of free water, while the incorporation of low-vapour-pressure cosolvents can reduce the overall tendency of the hydrogel to lose water [69]. Polyols, ionic liquids, and deep eutectic solvents can further strengthen interactions between water molecules and the polymer networks through hydrogen bonding, ion–dipole interactions, and hydration effects, thereby reducing the fraction of free water. Among these, polyols such as glycerol, ethylene glycol, and sorbitol have strong water-binding capacities and low vapour pressures, and are therefore widely used to obtain improved hydrogel’s water retention [82]. For example, Wang et al. replaced free water with a glycerol/water mixed solvent and introduced LiCl or Ca^2+^ ions to suppress water evaporation and ice formation at low temperature [83]. This strategy substantially improved the hydrogel’s resistance to dehydration and freezing, as well as its long-term environmental stability. Such solvent and ionic modifications provide an effective approach for maintaining stable sensing performance under prolonged use and challenging environmental conditions.

During extended operation, hydrogel-based mechanical sensors may undergo volume expansion due to moisture absorption or solvent exchange, resulting in polymer network rearrangement, disruption of conductive pathways, and output signal drift. To address this issue, strategies including hydrophobic association, hierarchical dynamic cross-linking, dual-network construction, and amphiphilic polymer design have been developed to regulate water transport and suppress excessive swelling, thereby improving dimensional stability [26,50]. For instance, a hydrophobic micellar system was constructed using sodium dodecyl sulfate, NaCl, and LMA. In this system, sodium dodecyl sulfate and NaCl induce the formation of stable micellar structures that encapsulate the hydrophobic monomer LMA, generating uniformly distributed hydrophobic microdomains. These hydrophobic domains serve as physical cross-linking points that reinforce the network structure while simultaneously limiting water penetration through hydrophobic interactions, thereby providing excellent swelling resistance [50]. Similarly, an amphiphilic copolymer network based on acrylamide and 2-hydroxyethyl methacrylate was reported to regulate hydration-induced swelling behavior through balanced hydrophilic and hydrophobic interactions, significantly improving dimensional stability and enabling reliable sleep apnoea monitoring under humid conditions [26].

Fatigue damage induced by long-term cyclic loading can also compromise sensor’s reliability. Researchers have enhanced the energy dissipation and damage recovery capabilities of hydrogels through the development of double-network architectures, multilevel dynamic cross-linking, and stress-dispersing structures, thereby improving fatigue resistance and cyclic stability [16,40]. For example, a fatigue-resistant ion gel incorporating multiple dynamic cross-links enables long-term and stable monitoring of respiratory abnormalities [16]. Furthermore, indentation-deformation triboelectric sensors have improved cycling stability and signal repeatability through rational structural optimization, providing a reliable platform for continuous sleep monitoring [40]. For sleep respiratory monitoring, fatigue resistance depends not only on the ability of a material to withstand repeated mechanical loading, but also on its capacity for structural recovery after deformation. Reversible physical interactions and dynamic cross-linked networks can dissipate mechanical energy through bond dissociation and reorganization, thereby reducing residual strain, and preserving structural integrity and stable signal output during prolonged cycling [17]. Furthermore, time-dependent mechanical behaviours, including stress relaxation, creep, and viscoelasticity, can significantly affect the long-term signal stability of hydrogel-based sensors. Stress relaxation refers to the gradual decrease in stress over time under sustained deformation, whereas creep describes the progressive deformation of a material under a constant load. Both phenomena contribute to the signal attenuation, hysteresis, and baseline drift during prolonged sensing [84]. Appropriate regulation of hydrogel viscoelasticity can help to balance mechanical compliance, structural stability, and dynamic responsiveness, thereby improving the sensor’s ability of reliably tracking continuous, low-strain, and low-frequency respiratory movements during sleep [85].

In addition to the intrinsic stability of the hydrogel, interfacial adhesion between the hydrogel and skin is a critical factor governing the reliability of long-term monitoring. To address this issue, various strategies, including mussel-inspired chemistry, physical interlocking, and ionic interactions, have been developed to enhance hydrogel–skin adhesion. Inspired by mussel adhesion mechanisms, catechol groups can establish multiple interfacial interactions through hydrogen bonding, coordination, and other non-covalent interactions, enabling strong and stable adhesion even on moist skin surfaces [86]. For example, Shao et al. employed multiple coordination interactions among TA, Cellulose nanocrystals, PAA chains, and metal ions to construct a tough, self-adhesive hydrogel [87]. The catechol groups provided by TA further enhanced skin adhesion, allowing the hydrogel to combine mechanical toughness, ionic conductivity, and a stable biointerface for pulse and respiration monitoring. Physical interlocking exploits surface microstructures, such as skin textures and mucosal folds, to provide anchoring sites. The hydrogel penetrates and conforms to microscopic surface irregularities, forming topological interlocking that substantially enhances interfacial adhesion strength [88]. Ionic bonding primarily relies on electrostatic attraction, ion pairing, and coordination between charged groups within the hydrogel and oppositely charged sites at the tissue interface to form reversible interfacial bonds. Because these ionic interactions are dynamic and reversible, they can reorganize in response to subtle skin deformations, thereby maintaining stable adhesion while accommodating flexible deformation [89].

However, high adhesion strength does not necessarily translate into superior long-term wear performance. Excessively strong interfacial bonding may increase mechanical irritation during removal, and restrict the natural deformation of the chest and abdomen. Therefore, interfacial design for long-term sleep monitoring should balance adhesive strength, reusability, skin comfort, and freedom of movement [90]. In addition, sweat, sebum, and hair under real-world skin conditions may alter interfacial wettability, effective contact area, and ionic interactions, thereby compromising hydrogel’s adhesion stability. Repeated application and removal may further cause interfacial contamination, depletion of adhesive groups, and the changes in surface morphology [91]. Consequently, future studies should comprehensively evaluate adhesion performance, interfacial slippage, and signal stability under varying levels of perspiration, humidity, and skin conditions. Combining cyclic adhesion–peeling and dynamic deformation tests would help to establish a systematic evaluation framework for long-term interfacial stability that more closely reflects the sleep scenarios.

### 3.4. High Wearing Comfort

Conventional hydrogels suffer from issues such as high modulus, insufficient breathability, and a tendency to cause skin stuffiness and sweat accumulation during prolonged wear. By regulating polymer network density, introducing flexible polymer segments, and constructing porous architectures, the mechanical properties of hydrogels can be tailored to better match the softness and deformability of biological tissues. These approaches reduce perceived pressure and interfacial stress during wear, thereby improving comfort during long-term monitoring [92,93].

In addition to mechanical adaptation, enhancing moisture and thermal transport capabilities is essential for maintaining skin comfort during continuous operation. The construction of macroporous network structures can effectively facilitate water vapor diffusion and heat dissipation while improving the overall breathability of hydrogel-based mechanical sensors. For example, an interpenetrating macroporous network was developed through a “lock–unlock” in situ salting-out phase-separation strategy combined with functionalization with deep eutectic solvents. This structural design not only improves water vapor and heat transport efficiency, and reduces the effective modulus of the hydrogel, but also provides excellent breathability, moisture retention, and environmental stability. As a result, the sensor achieves improved long-term wearing comfort while maintaining reliable respiratory monitoring performance [7].

## 4. Conclusions and Outlook

Hydrogel-based mechanical sensors demonstrate significant potential for sleep apnoea monitoring owing to their flexibility, stretchability, biocompatibility, and excellent mechanical compatibility with human tissues. In recent years, various conductive hydrogels, including ion-modified hydrogels, metal-based hydrogels, carbon-based hydrogels, and MXene-based hydrogels, are proposed and investigated. When combined with multimodal sensing, structural optimization, and intelligent algorithms, these systems enable highly sensitive and continuous monitoring of respiratory status, apnoea events, and multi-parameter physiological signals, providing a promising technical approach for the early screening of sleep-related respiratory disorders. However, hydrogel-based mechanical sensors still face challenges in terms of long-term stability, accuracy, comfort, and clinical applications. Signal drift caused by body movements and environmental variations, dehydration and fatigue damage during long-term use, and limited multimodal signal fusion capabilities continue to restrict their practical implementation. Future investigation should focus on the development of highly stable hydrogels with enhanced dehydration, freeze, and fatigue resistance, and tissue-matched low-modulus properties, while integrating multimodal sensing, artificial intelligence analysis, and flexible wireless integration technologies to further improve the accuracy, intelligence, and clinical applicability of sleep-related respiratory monitoring.

## Figures and Tables

**Table 1:** Comparison of hydrogel-based mechanical sensors for sleep respiratory monitoring and their performance characteristics.

Materials	Fabrication Strategies	Sensing Mechanisms	Sensitivity	Response Time	Cyclic Stability	Detection Range	Structural Features	Application Scenarios	References
Ion-modified hydrogel	Freeze–thaw cycling + chemical cross-linking + ion immersion	Piezoresistive	GF = 0.79 (0–370%)	0.26 s	20,000 cycles (30%)	0–370%	Anisotropic collagen-fibre structure	Monitoring of sleep-related snoring and chronic respiratory diseases (chest and abdominal respiration)	[16]
Ion-modified hydrogel	Chemical cross-linking + ion immersion	Capacitive and piezoresistive	0.053 kPa^−1^ (0–45 kPa); 0.01 kPa^−1^ (45–300 kPa)	0.02 s	15 days (2.5 kPa)	1.25–10 kPa (−40 °C)	Dual-network sandwich structure	Sleep apnoea monitoring (nasal airflow, thoracic/abdominal movement and pulse)	[21]
Ion-modified hydrogel	Freeze–thaw cycling + ion immersion	Piezoresistive and capacitive	/	/	1000 cycles	0–400%	Egg-box structure	Obstructive sleep apnoea monitoring (abdominal movement and nasal airflow)	[22]
Ion-modified hydrogel	In situ polymerization + chemical cross-linking + in situ compositing	Piezoresistive	GF = 4.11; GF = 0.86 (0–100%)	0.1 s	50 cycles (100%)	0–1000%	Bio-based flexible network	Sleep respiratory monitoring and human–machine interaction (chest movement)	[24]
Ion-modified hydrogel	Freeze–thaw cycling + in situ compositing	Triboelectric	7.42 V kPa^−1^ (0.248–1.26 kPa); 1.53 V kPa^−1^ (1.26–27.56 kPa); 0.14 V kPa^−1^ (27.56–87.4 kPa)	0.022 s	100,000 cycles (60 kPa)	0.248–87.4 kPa	Sandwich structure	Obstructive sleep apnoea–hypopnoea syndrome monitoring (wrist and back)	[40]
Ion-modified hydrogels	In situ polymerization + chemical cross-linking + in situ compositing	Piezoresistive	GF = 1.12 (1–500%); GF = 1.77 (500–1000%); GF = 2.13 (1000–1500%)	0.4 s	1000 cycles (100%)	1–1500%	Hydrophobic micellar structure; multifunctional dynamic network	Monitoring of sleep-related breathing disorders (rapid, slow, and normal breathing; chest, abdomen, and throat)	[50]
Metal-composite hydrogel	In situ polymerization + chemical cross-linking + in situ compositing	Piezoresistive	GF = 2.20 (0–100%); GF = 4.40 (100–200%); GF = 6.73 (200–350%); GF = 8.98 (350–500%)	0.139 s	200 cycles (50%)	1–500%	Dynamic cross-linking network	Sleep snoring monitoring (throat) and sleep eye movement monitoring (eyelids)	[17]
Metal-composite hydrogel	Freeze–thaw cycling + ionic immersion	Piezoresistive	GF = 0.61 (strain < 50%); GF = 0.54 (strain > 50%)	0.21 s	1000 cycles	0–300%	Dual-network reinforced structure	Sleep apnoea monitoring (chest movement); intelligent alarm function	[19]
Carbon-based/metal-composite hydrogel	In situ polymerization + in situ compositing	Piezoresistive	GF = 0.51 (0–200%); GF = 0.75 (200–840%)	0.11 s	500 cycles (10%)	0–840%	Dual conductive pathways; amphiphilic copolymer network	Sleep apnoea monitoring (chest movement)	[26]
Carbon-based composite hydrogel	Electrostatic flocking + alkaline treatment	Piezoresistive	GF = 20.1 (0–7%); GF = 36.9 (7–13%); GF = 76.1 (13–20%)	Airflow response time: 0.10 s; tensile response time: 0.6 s	Airflow: 400 cycles (0.199 m s^−1^); tensile: 250 cycles (2% strain)	0–20%	Skin- and hair-inspired nanofibre structure	Sleep apnoea detection (nasal cannula, breathing mask and abdominal respiration); gesture recognition and communication (knuckle motion)	[27]
MXene composite + ion-modified hydrogel	In situ polymerization + in situ compositing	Piezoresistive	GF = 5.12 (0–50%); GF = 23.31 (60–180%)	0.198 s	500 cycles (0–150%)	0–180%	Island–bridge structure	Comprehensive sleep monitoring (chest and abdominal respiration)	[72]
MXene composites + ion-modified hydrogels	In situ polymerization + chemical cross-linking + in situ compositing	Piezoresistive and piezoelectric	/	0.165 s	500 tensile cycles (100%); 3000 compression cycles (70%)	/	Dynamic cross-linking network	Sleep respiratory monitoring (nasal, oral, thoracic, and abdominal respiration)	[45]

## Data Availability

No new data were created or analyzed in this study. Data sharing is not applicable to this article.

## Abbreviations

The following abbreviations are used in this manuscript:

OSA	Obstructive sleep apnoea
PVA	Polyvinyl alcohol
SF	Silk fibroin
CSN	Conductive and stretchable nanostructured
3D	Three-dimensional
PAM	Polyacrylamide
TENGs	Triboelectric nanogenerators
PEGDA	Poly(ethylene glycol) diacrylate
BS-TENG	Biodegradable single-electrode triboelectric nanogenerator
rGO	Reduced graphene oxide
CFs	Arbon fibres
APS	Ammonium persulfate
CS	Chitosan
PACG	Poly(N-acryloyl-2-glycine)
LMA	Lauryl methacrylate
CMC	Carboxymethyl cellulose

## References

[1] Cowie M.R., Linz D., Redline S., Somers V.K., Simonds A.K. (2021). Sleep Disordered Breathing and Cardiovascular Disease: JACC State-of-the-Art Review. J. Am. Coll. Cardiol..

[2] Veasey Sigrid C., Rosen Ilene M. (2019). Obstructive Sleep Apnea in Adults. N. Engl. J. Med..

[3] Steelman E., Reid E., Palomino M., DelRosso L. (2026). 0542 Accuracy and Predictive Value of a PAD Sleep Monitoring Device Compared with In-Lab Polysomnography. Sleep.

[4] Wang J., Xu J., Ma J., Gong J., Ji L., Guo W., Yue H., Qiao Y., Zhou J. (2026). Wearable Sensors for Monitoring Abnormalities in Sleep-Related Breathing. ACS Appl. Mater. Interfaces.

[5] Sun X., Yao F., Li J. (2020). Nanocomposite hydrogel-based strain and pressure sensors: A review. J. Mater. Chem. A.

[6] Li Z., Chen L., Liu F., Liu X. (2025). Chitosan-based hydrogels with stretchable, self-healing, self-adhesive properties for flexible sensing applications. Colloids Surf. A Physicochem. Eng. Asp..

[7] Wang R., Cai X., You L., Chen G., Wang S. (2026). A locking-unlocking strategy enables open macroporous hydrogels for diagnosing-treating apnea. Chem. Eng. J..

[8] Li Z., Zhu J., Wang Z., Hu H., Zhang T. (2025). Dual-Responsive Starch Hydrogels via Physicochemical Crosslinking for Wearable Pressure and Ultra-Sensitive Humidity Sensing. Sensors.

[9] Zhang Y., Xu Z., Yuan Y., Liu C., Zhang M., Zhang L., Wan P. (2023). Flexible Antiswelling Photothermal-Therapy MXene Hydrogel-Based Epidermal Sensor for Intelligent Human–Machine Interfacing. Adv. Funct. Mater..

[10] Deng S., Ming X., Yan G., Wang L., Li Z., Chen J., Lai J., Li D., Xiang D., Zhao C. (2024). Double-network conductive hydrogel for non-contact respiratory monitoring. Chem. Eng. J..

[11] Wang L., Xu T., Zhang X. (2021). Multifunctional conductive hydrogel-based flexible wearable sensors. TrAC Trends Anal. Chem..

[12] Li Q., Tian B., Liang J., Wu W. (2023). Functional conductive hydrogels: From performance to flexible sensor applications. Mater. Chem. Front..

[13] Han M., Luo D., Talha K., He J., Xing M., Chen L., Liu H. (2025). Research progress onconductive hydrogels and their applications in flexible sensors: A review. J. Mater. Chem. A.

[14] Li X., Gong J.P. (2024). Design principles for strong and tough hydrogels. Nat. Rev. Mater..

[15] Fang Y.-H., Liang C., Liljeström V., Lv Z.-P., Ikkala O., Zhang H. (2024). Toughening Hydrogels with Fibrillar Connected Double Networks. Adv. Mater..

[16] Zha X.-J., Li J.-B., Liang G.-P., Pu J.-H., Zhang Z.-W., Wang B., Huang J.-G., Jia J., Zhao X., Pan K.-Q. (2023). Anti-fatigue ionic gels for long-term multimodal respiratory abnormality monitoring. J. Mater. Sci. Technol..

[17] Wang J., Du P., Hsu Y.-I., Uyama H. (2024). Rapid preparation of dynamic-crosslinked nanocomposite hydrogel sensors with efficiency self-healing and adhesion properties for elderly health and sleep management. Chem. Eng. J..

[18] Dhand A.P., Galarraga J.H., Burdick J.A. (2021). Enhancing Biopolymer Hydrogel Functionality through Interpenetrating Networks. Trends Biotechnol..

[19] Yan H., Wei L., Shang K., Weng Q., Lei Y., Cheng J., Sun Q., Kim D.-H., Lin X. (2026). Mechanically Enhanced, Antibacterial, and Double-Network Hydrogel Flexible Sensors for Sleep Apnea Monitoring. Small.

[20] Fan Z., Ji D., Kim J. (2023). Recent Progress in Mechanically Robust and Conductive-Hydrogel-Based Sensors. Adv. Intell. Syst..

[21] Liu J., Wang H., Liu T., Wu Q., Ding Y., Ou R., Guo C., Liu Z., Wang Q. (2022). Multimodal Hydrogel-Based Respiratory Monitoring System for Diagnosing Obstructive Sleep Apnea Syndrome. Adv. Funct. Mater..

[22] Liu J., Zhao W., Li J., Li C., Xu S., Sun Y., Ma Z., Zhao H., Ren L. (2024). Multimodal and flexible hydrogel-based sensors for respiratory monitoring and posture recognition. Biosens. Bioelectron..

[23] Han S., Li S., Fu X., Han S., Chen H., Zhang L., Wang J., Sun G. (2024). Research Progress of Flexible Piezoresistive Sensors Based on Polymer Porous Materials. ACS Sens..

[24] Liu R., Wang Y., Chu H., Li Y., Li Y., Zhao Y., Tian Y., Xia Z. (2024). High-performance gelatin-based hydrogel flexible sensor for respiratory monitoring and human–machine interaction. Chem. Eng. J..

[25] Zhang Y.-Z., Lee K.H., Anjum D.H., Sougrat R., Jiang Q., Kim H., Alshareef H.N. (2018). MXenes stretch hydrogel sensor performance to new limits. Sci. Adv..

[26] Tian R., Zhang S., Shi F., Lu S., Liu Y., Deng H., Jiang Y., Sun Y. (2025). Molecularly synergistic organogel with engineered conductivity and anti-swelling unity for reliable sleep apnea monitoring. Chem. Eng. J..

[27] Shen M., Ren Z., Huang H., Lin T., Fu W., Cheng S., Liu J., Cheng S. (2025). Skin- and hair- inspired nanofibrious hydrogel composite as multimodal sensor for polysomnographic monitoring in sleep apnea syndrome. J. Colloid Interface Sci..

[28] Chen Z., Wang Z., Li X., Lin Y., Luo N., Long M., Zhao N., Xu J.-B. (2017). Flexible Piezoelectric-Induced Pressure Sensors for Static Measurements Based on Nanowires/Graphene Heterostructures. ACS Nano.

[29] Chen K., Liu B., Hu N., Fan Q., Zhan F., Zhang Z., Ni Z., Li X., Hu T. (2024). A biodegradable, highly sensitive and multifunctional mechanical sensor based on rGO-silk fibroin hydrogel for human motion detection and gesture recognition. J. Mater. Chem. A.

[30] He X., Zhang B., Liu Q., Chen H., Cheng J., Jian B., Yin H., Li H., Duan K., Zhang J. (2024). Highly conductive and stretchable nanostructured ionogels for 3D printing capacitive sensors with superior performance. Nat. Commun..

[31] Wei Q., Gao W., Zhang T., Long L., Zhao J., Pan L., Yuan X. (2026). A “PHASE” Strategy for Preparing Flexible Piezoelectric Polylactic Acid Double Network Hydrogel Sensors. Adv. Funct. Mater..

[32] Li Z., Li C., Sun W., Bai Y., Li Z., Deng Y. (2023). A Controlled Biodegradable Triboelectric Nanogenerator Based on PEGDA/Laponite Hydrogels. ACS Appl. Mater. Interfaces.

[33] Sun H., Sun Y., Buranabunwong C., Li X., Zhang S., Chen Y., Li M. (2023). Flexible capacitive sensor based on Miura-ori structure. Chem. Eng. J..

[34] Jia X., Xu Z., Huang J., Zhu Y., Xi S., Zhang J., Wang X. (2026). Flexible Capacitive Pressure Sensors with Ultrasonically Engineered Cu-Filled PDMS Dielectric Layers. Sensors.

[35] Motaghedi F., Rose L., Wu Y., Carmichael R.S., Ahamed M.J., Rondeau-Gagné S., Carmichael T.B. (2025). All-Textile Wearable Capacitive Pressure Sensors Based on Cut-Pile Fabrics with Integrated Electrodes. ACS Appl. Mater. Interfaces.

[36] Mo F., Huang Y., Li Q., Wang Z., Jiang R., Gai W., Zhi C. (2021). A Highly Stable and Durable Capacitive Strain Sensor Based on Dynamically Super-Tough Hydro/Organo-Gels. Adv. Funct. Mater..

[37] Lu J., Hu S., Li W., Wang X., Mo X., Gong X., Liu H., Luo W., Dong W., Sima C. (2022). A Biodegradable and Recyclable Piezoelectric Sensor Based on a Molecular Ferroelectric Embedded in a Bacterial Cellulose Hydrogel. ACS Nano.

[38] Yin J., Jia P., Ren Z., Zhang Q., Lu W., Yao Q., Deng M., Zhou X., Gao Y., Liu N. (2023). Recent Advances in Self-Powered Sensors Based on Ionic Hydrogels. Research.

[39] Dobashi Y., Yao D., Petel Y., Nguyen T.N., Sarwar M.S., Thabet Y., Ng C.L.W., Scabeni Glitz E., Nguyen G.T.M., Plesse C. (2022). Piezoionic mechanoreceptors: Force-induced current generation in hydrogels. Science.

[40] Lu B., Xie L., Lei H., Liu Y., Ren Y., SiMa Z., Gu H., Wang Y., Ji H., Shi J. (2025). Indentation deformation-based triboelectric pressure sensor for high accurate sleep monitoring. Chem. Eng. J..

[41] Wu Y., Luo Y., Cuthbert T.J., Shokurov A.V., Chu P.K., Feng S.-P., Menon C. (2022). Hydrogels as Soft Ionic Conductors in Flexible and Wearable Triboelectric Nanogenerators. Adv. Sci..

[42] Cui Y., Yang T., Luo H., Zheng Y. (2026). Triboelectric Nanogenerators Promote Self-Powered Sensing and Intelligent Monitoring. Sensors.

[43] Yang Q., Yu M., Zhang H., Li N., Du J., Xu L., Xu J. (2025). Triboelectric nanogenerator based on well-dispersed and oxide-free liquid metal-doped conductive hydrogel as self-powered wearable sensor for respiratory and thyroid cartilage signal monitoring. Nano Energy.

[44] Zha X.-J., Li Q.-K., Chen P., Liu Y., Huang J., Li J.-B., Zhou Y. (2026). Flexible and multimodal sensing platforms for wearable respiratory monitoring: Innovations in materials and systems for precision health. Chem. Eng. J..

[45] Ni Y., Zang X., Yang Y., Gong Z., Li H., Chen J., Wu C., Huang J., Lai Y. (2024). Environmental Stability Stretchable Organic Hydrogel Humidity Sensor for Respiratory Monitoring with Ultrahigh Sensitivity. Adv. Funct. Mater..

[46] Lu Y., Yang G., Wang S., Zhang Y., Jian Y., He L., Yu T., Luo H., Kong D., Xianyu Y. (2024). Stretchable graphene–hydrogel interfaces for wearable and implantable bioelectronics. Nat. Electron..

[47] Huang J., Huang X., Wu P. (2024). Readily prepared and processed multifunctional MXene nanocomposite hydrogels for smart electronics. SmartMat.

[48] Ni Y., Li S., Yin S., Li M., Yang Y., Huang J., Lai Y., Parkin I.P., Carmalt C.J., Li H. (2026). Ionic Conductive Organohydrogel with Multi-Environmental Stability for Machine Learning-Assisted Health Monitoring. Adv. Funct. Mater..

[49] Liu Y., Wang C., Xue J., Huang G., Zheng S., Zhao K., Huang J., Wang Y., Zhang Y., Yin T. (2022). Body Temperature Enhanced Adhesive, Antibacterial, and Recyclable Ionic Hydrogel for Epidermal Electrophysiological Monitoring. Adv. Healthc. Mater..

[50] Zhao Y., Wang Z., Wang Y., Cheng Z. (2026). Mechanically resilient, freeze-resistant, and antibacterial carboxymethyl cellulose/phytic acid hydrogel sensors for respiratory monitoring. Int. J. Biol. Macromol..

[51] Zhang B., Cai X., Tian S., Liang J., Helal M.H., Alshammari D.A., El-Bahy Z.M., Lu B., Zhao J., Zhou J. (2025). Zwitterionic Hydrogels Endow Zinc-Ion Micro-Batteries with Superior Durability for Electrophysiological Monitoring. Adv. Energy Mater..

[52] Guan X., Ma X., Gao R., Zheng Q., Sun C., Chen Y., Mu J. (2026). Low-Temperature One-Pot Fabrication of a Dual-Ion Conductive Hydrogel for Biological Monitoring. Sensors.

[53] Roy A., Afshari R., Jain S., Zheng Y., Lin M.-H., Zenkar S., Yin J., Chen J., Peppas N.A., Annabi N. (2025). Advances in conducting nanocomposite hydrogels for wearable biomonitoring. Chem. Soc. Rev..

[54] Rao N., Singh R., Bashambu L. (2021). Carbon-based nanomaterials: Synthesis and prospective applications. Mater. Today Proc..

[55] Walker B.W., Portillo Lara R., Mogadam E., Hsiang Yu C., Kimball W., Annabi N. (2019). Rational design of microfabricated electroconductive hydrogels for biomedical applications. Prog. Polym. Sci..

[56] Sun X., Qin Z., Ye L., Zhang H., Yu Q., Wu X., Li J., Yao F. (2020). Carbon nanotubes reinforced hydrogel as flexible strain sensor with high stretchability and mechanically toughness. Chem. Eng. J..

[57] Parajuli D., Murali N., K. C. D., Karki B., Samatha K., Kim A.A., Park M., Pant B. (2022). Advancements in MXene-Polymer Nanocomposites in Energy Storage and Biomedical Applications. Polymers.

[58] Wang B., Zhou L., Wei S., Zhu Q., Wu Q., Cao C. (2026). Nacre-Inspired Flexible MXene-Based Films for Multifunctional Applications in Supercapacitors and Piezoresistive Sensors. Sensors.

[59] Khan K., Tareen A.K., Iqbal M., Ye Z., Xie Z., Mahmood A., Mahmood N., Zhang H. (2023). Recent Progress in Emerging Novel MXenes Based Materials and their Fascinating Sensing Applications. Small.

[60] Iravani S., Rabiee N., Makvandi P. (2024). Advancements in MXene-based composites for electronic skins. J. Mater. Chem. B.

[61] Li Y., Yang D., Wu Z., Gao F.-L., Gao X.-Z., Zhao H.-Y., Li X., Yu Z.-Z. (2023). Self-adhesive, self-healing, biocompatible and conductive polyacrylamide nanocomposite hydrogels for reliable strain and pressure sensors. Nano Energy.

[62] Yu H., Yang X., Chen J., Liu L., Li J., Yang C., Jiao G., Sun L., Chen Z. (2026). Hydrogel Electronic Skin Synergistically Enhanced by Multibond Crosslinking and a Negative Poisson’s Ratio Structure for Human Respiration Monitoring. ACS Sens..

[63] Ma X., Li Y., Wang W., Ji Q., Xia Y. (2013). Temperature-sensitive poly(N-isopropylacrylamide)/graphene oxide nanocomposite hydrogels by in situ polymerization with improved swelling capability and mechanical behavior. Eur. Polym. J..

[64] Luo J., Jin Y., Li L., Chang B., Zhang B., Li K., Li Y., Zhang Q., Wang H., Wang J. (2024). A selective frequency damping and Janus adhesive hydrogel as bioelectronic interfaces for clinical trials. Nat. Commun..

[65] Zhang C., Lu H., Wang X. (2022). Transient Polymer Hydrogels Based on Dynamic Covalent Borate Ester Bonds. Chin. J. Chem..

[66] Nugent M.J.D., Higginbotham C.L. (2007). Preparation of a novel freeze thawed poly(vinyl alcohol) composite hydrogel for drug delivery applications. Eur. J. Pharm. Biopharm..

[67] Lu J., Hu O., Hou L., Ye D., Weng S., Jiang X. (2022). Highly tough and ionic conductive starch/poly(vinyl alcohol) hydrogels based on a universal soaking strategy. Int. J. Biol. Macromol..

[68] Di X., Ma Q., Xu Y., Yang M., Wu G., Sun P. (2021). High-performance ionic conductive poly(vinyl alcohol) hydrogels for flexible strain sensors based on a universal soaking strategy. Mater. Chem. Front..

[69] Fang L., Zhang J., Wang W., Zhang Y., Chen F., Zhou J., Chen F., Li R., Zhou X., Xie Z. (2020). Stretchable, Healable, and Degradable Soft Ionic Microdevices Based on Multifunctional Soaking-Toughened Dual-Dynamic-Network Organohydrogel Electrolytes. ACS Appl. Mater. Interfaces.

[70] Dou X., Wang H., Yang F., Shen H., Wang X., Wu D. (2023). One-Step Soaking Strategy toward Anti-Swelling Hydrogels with a Stiff “Armor”. Adv. Sci..

[71] Vijayakumar V., Anothumakkool B., Kurungot S., Winter M., Nair J.R. (2021). In situ polymerization process: An essential design tool for lithium polymer batteries. Energy Environ. Sci..

[72] Liu X., Zhang R., Wang D., Meng C. (2026). A Self-Adhesive, Motion-Resistant Multifunctional Hydrogel Patch with Island-Bridge Design for Comprehensive Sleep Monitoring. ACS Appl. Mater. Interfaces.

[73] Xu H., Hao Z., Wang C., Deng J., Wang T., Zhang J. (2023). In Situ Emulsion Polymerization to Multifunctional Polymer Nanocomposites: A Review. Macromol. Chem. Phys..

[74] Maqbool Q., Holten-Andersen N. (2024). In Situ Mineralization in Metal-Ion-Coordinated Polymer Hydrogels: Progress, Challenges, and Opportunities. Chem. Mater..

[75] Luo J., Ji N., Zhang W., Ge P., Liu Y., Sun J., Wang J., Zhuo Q., Qin C., Dai L. (2022). Ultrasensitive airflow sensor prepared by electrostatic flocking for sound recognition and motion monitoring. Mater. Horiz..

[76] Shen C., Sun S., Zhang H., Zhang Z. (2023). Bioinspired Ultrasensitive and Flexible Airflow Sensor based on Short Carbon Fiber Network. Adv. Mater. Technol..

[77] Shen M., Deng S., Pi H., Ren Z., Zhu Y., Ikkala O., Zhang H., Qin C., Cheng S. (2025). Electrostatic Flocking: Reborn to Embrace Multifunctional Applications. Small Struct..

[78] Chen S., Zhang Y., Li Y., Wang P., Hu D. (2024). Recent development of flexible force sensors with multiple environmental adaptations. Nano Energy.

[79] Yuan Y., Zhang Q., Lin S., Li J. (2025). Water: The soul of hydrogels. Prog. Mater. Sci..

[80] Zhao X., Shi A., Shi L., Sun W., You Z. (2025). An Overview of Recent Advances in Antidehydration and Antifreezing Hydrogels. ACS Appl. Energy Mater..

[81] Chang Y., Jia Y., Pan Y., Wang J., Yang H., Zu M., Cheng H. (2026). Enhancing water retention in hydrogels under extreme conditions: Strategies, applications and challenges. Mater. Sci. Eng. R Rep..

[82] Li M., Wang X., Dong B., Sitti M. (2020). In-air fast response and high speed jumping and rolling of a light-driven hydrogel actuator. Nat. Commun..

[83] Wang X.-Y., Kim H.-J. (2022). Ultra-stretchable dual-network ionic hydrogel strain sensor with moistening and anti-freezing ability. Prog. Org. Coat..

[84] Qiu H.N., Lin J., Hou L.X., Xiao R., Zheng Q., Wu Z.L. (2025). Stress Relaxation and Creep Response of Glassy Hydrogels with Dense Physical Associations. ACS Appl. Mater. Interfaces.

[85] Lin Y.-H., Lou J., Xia Y., Chaudhuri O. (2024). Cross-Linker Architectures Impact Viscoelasticity in Dynamic Covalent Hydrogels. Adv. Healthc. Mater..

[86] Xie C., Wang X., He H., Ding Y., Lu X. (2020). Mussel-Inspired Hydrogels for Self-Adhesive Bioelectronics. Adv. Funct. Mater..

[87] Shao C., Wang M., Meng L., Chang H., Wang B., Xu F., Yang J., Wan P. (2018). Mussel-Inspired Cellulose Nanocomposite Tough Hydrogels with Synergistic Self-Healing, Adhesive, and Strain-Sensitive Properties. Chem. Mater..

[88] Sun H., Qu X., Wang Q., Guo Y., Dong X. (2026). Dynamic regulation of interfacial adhesion in biomedical hydrogels. Chem. Soc. Rev..

[89] Bovone G., Dudaryeva O.Y., Marco-Dufort B., Tibbitt M.W. (2021). Engineering Hydrogel Adhesion for Biomedical Applications via Chemical Design of the Junction. ACS Biomater. Sci. Eng..

[90] Wang S., Wang Z., Zhang L., Song Z., Liu H., Xu X. (2024). Sweat-adaptive adhesive hydrogel electronics enabled by dynamic hydrogen bond networks. Chem. Eng. J..

[91] Zhang W., Wang R., Sun Z., Zhu X., Zhao Q., Zhang T., Cholewinski A., Yang F., Zhao B., Pinnaratip R. (2020). Catechol-functionalized hydrogels: Biomimetic design, adhesion mechanism, and biomedical applications. Chem. Soc. Rev..

[92] Wang Y., Gao Y., Tang L., Guo Y., Sha B., Jiang Y. (2026). Hydrogel-based wearable and implantable biosensors in health monitoring. Biomater. Sci..

[93] Kim D., Bang J., Jeong J., Koo D.L., Ko S.H. (2025). Intelligent soft wearable bioelectronics for neurological disorders. Mater. Horiz..

